# A Role for the Budding Yeast Separase, Esp1, in Ty1 Element Retrotransposition

**DOI:** 10.1371/journal.pgen.1005109

**Published:** 2015-03-30

**Authors:** Krystina L. Ho, Lina Ma, Stephanie Cheung, Savrina Manhas, Nancy Fang, Kaiqian Wang, Barry Young, Christopher Loewen, Thibault Mayor, Vivien Measday

**Affiliations:** 1 Department of Biochemistry and Molecular Biology, University of British Columbia, Vancouver, British Columbia, Canada; 2 Wine Research Centre, University of British Columbia, Vancouver, British Columbia, Canada; 3 Centre for High-Throughput Biology, University of British Columbia, Vancouver, British Columbia, Canada; 4 Department of Cellular and Physiological Sciences, University of British Columbia, Vancouver, British Columbia, Canada; Johns Hopkins School of Medicine, UNITED STATES

## Abstract

Separase/Esp1 is a protease required at the onset of anaphase to cleave cohesin and thereby enable sister chromatid separation. Esp1 also promotes release of the Cdc14 phosphatase from the nucleolus to enable mitotic exit. To uncover other potential roles for separase, we performed two complementary genome-wide genetic interaction screens with a strain carrying the budding yeast *esp1-1* separase mutation. We identified 161 genes that when mutated aggravate *esp1-1* growth and 44 genes that upon increased dosage are detrimental to *esp1-1* viability. In addition to the expected cell cycle and sister chromatid segregation genes that were identified, 24% of the genes identified in the *esp1-1* genetic screens have a role in Ty1 element retrotransposition. Retrotransposons, like retroviruses, replicate through reverse transcription of an mRNA intermediate and the resultant cDNA product is integrated into the genome by a conserved transposon or retrovirus encoded integrase protein. We purified Esp1 from yeast and identified an interaction between Esp1 and Ty1 integrase using mass spectrometry that was subsequently confirmed by co-immunoprecipitation analysis. Ty1 transposon mobility and insertion upstream of the *SUF16* tRNA gene are both reduced in an *esp1-1* strain but increased in cohesin mutant strains. Securin/Pds1, which is required for efficient localization of Esp1 to the nucleus, is also required for efficient Ty1 transposition. We propose that Esp1 serves two roles to mediate Ty1 transposition – one to remove cohesin and the second to target Ty1-IN to chromatin.

## Author Summary

Separases are a family of cysteine proteases found in organisms ranging from yeast to humans that are required for separation of chromosomes during cell division. Separases dissolve the cohesin ring-like complex that holds sister chromatids together until chromosome separation occurs during mitosis. We used two genetic screens in the model organism budding yeast to identify additional cellular roles for separase. Surprisingly, we found that yeast separase is required for insertion of Ty1 retrotransposons into the yeast genome. Ty1 retrotransposons, or elements, are similar in their life cycle to retroviruses such as the human immunodeficiency virus type 1 (HIV-1) which is the cause of acquired immunodeficiency syndrome (AIDS). The insertion of retroviral/retrotransposon DNA into the genome requires a conserved protein encoded by the virus/retrotransposon called integrase. Until now, it was unknown which yeast host protein interacted with Ty1 integrase. We found that yeast separase interacts with Ty1 integrase, and that separase may be required for targeting Ty1 integrase into the genome via its interaction with integrase and by removing cohesin from the chromosomes. Due to the conservation of Ty1 integrase with other viral integrases, our discovery may shed light on how other viral integrases are targeted into the genome.

## Introduction

Successful mitotic cell division is predicated upon the faithful propagation of genetic material. A critical element to the regulation of this process is the use of cohesin, a proteinaceous ring-like structure, to pair sister chromatids [1]. This coupling ensures that chromosomal separation does not occur prior to successful bipolar attachment, thereby preventing chromosomal misseggregation. Sister chromatid separation is triggered by an evolutionarily conserved cysteine protease known as separase that cleaves cohesin—an event that is controlled by inhibiting the catalytic activity of these aptly named proteases by securin [2].

In *Saccharomyces cerevisiae*, the core cohesin complex consists of two sister chromatid cohesin (Scc1/Mcd1 and Scc3) and two structural maintenance of chromosome (Smc1 and Smc3) proteins [1]. Smc1 and Smc3 have a dimerization domain located in the middle of a coiled-coil region that folds back on itself and ends in two globular ATPase domains. The ATPase heads of Smc1 and Smc3 interact with Scc1 to form a ring-like complex whereas Scc3 interacts with Scc1. The cohesin ring loads onto chromosomes at sites of RNA polymerase III (RNA Pol III) transcription, dependent on the Scc2/4 cohesin loading complex, then moves to sites of RNA Pol II convergent transcription [3,4]. Securin, or Pds1, is targeted for proteolytic degradation at the onset of anaphase by the anaphase promoting complex, an E3 ubiquitin ligase that functions in conjunction with the co-activator Cdc20 [5,6]. Pds1 degradation frees the budding yeast separase, Esp1, to specifically target phosphorylated, chromatin-bound Scc1 for cleavage [7–9]. Cohesin can also be removed from chromosomes by a second separase-independent mechanism that occurs throughout the cell cycle. This so-called “anti-establishment” activity requires Scc3 and two accessory proteins that interact with the cohesin complex called Rad61 and Pds5 [10,11].

In higher eukaryotes, separases are implicated in roles in addition to cohesin cleavage such as centrosome duplication in humans [12], cell polarity in *Arabidopsis thaliana* [13] and epithelial re-organization in *Drosophila melanogaster* [14]. Similarly, Esp1 has demonstrable functions aside from cleaving Scc1, with the most extensively studied role focusing on Slk19, a kinesin-associated protein that is also cleaved by Esp1. By cleaving Slk19 during anaphase spindle elongation, Esp1 is thought to stabilize the anaphase spindle [15]. As well, both Esp1 and Slk19 serve to promote mitotic exit. Key to the initiation of this event is the biphasic release of the Cdc14 phosphatase from its sequestration in the nucleolus [16]. The initial wave of release is known as the Cdc14 early anaphase release (FEAR) pathway, with a second more prolonged release referred to as the mitotic exit network (MEN) [16,17]. Esp1 and Slk19 both function in the FEAR pathway, a role not dependent on the catalytic activity of Esp1 [18,19].

To gain insight into the cellular functions of Esp1, we undertook two genome-wide screens for Esp1 genetic interactions. Using synthetic genetic array (SGA) methodology, we investigated both synthetic lethal (SL) and synthetic dosage lethal (SDL) relationships of the *esp1-1* temperature sensitive mutant [20]. Analysis of *esp1-1* genetic interactions revealed that genes involved in the regulation of Ty1 transposition are enriched in the screens.

Ty retrotransposons are transposable elements that share similarities to retroviruses, though they lack an infectious phase in their life cycle. There are five families of Ty elements in *S*. *cerevisiae* (Ty1-5), the most abundant of which is Ty1 with 32 full size elements in the S288C genome. Each Ty element is flanked by long terminal repeats and is comprised of both TyA and TyB open reading frames (ORFs), analogous to retroviral *gag* and *pol* respectively. TyA encodes the structural elements of Gag (coat protein) while TyB is a polyprotein that is cleaved to generate a protease (PR), integrase (IN) and reverse transcriptase (RT) [21,22]. Ty1 elements insert upstream of genes transcribed by RNA pol III, such as tRNAs genes, but the host factors required for targeting Ty1 elements to the genome are still unclear. However there is precedent for Ty-IN specific host factors because Ty5-IN interacts with Sir4 to mediate insertion into silenced regions of the genome whereas Ty3-IN is targeted by the RNA Pol III TFIIIB factors Brf1 and TBP [23,24]. We have discovered that Esp1 specifically interacts with Ty1-IN and that *esp1* and *pds1* mutants have defects in Ty1 transposition whereas cohesin mutants have increased transposition events. We propose that both removal of cohesin by Esp1 and the physical interaction of Esp1 with Ty1-IN promote efficient Ty1 element insertion into the genome.

## Results

### Identification of esp1-1 genetic interactions

To uncover potential new roles for Esp1 using genome-wide genetic interaction studies, we subjected the *ESP1* temperature sensitive mutant, *esp1-1*, to SGA analysis [25]. We first sequenced the entire 4893 base pair (bp) ORF of *esp1-1* to determine the location of the relevant mutation(s). Fortuitously, we identified a single point mutation at bp 4211 (C→T), resulting in conversion of proline 1404 to leucine. This mutation is in the vicinity of the highly conserved histidine 1505 and cysteine 1531 residues of the Esp1 catalytic site [9]. The *esp1-1* mutant has significantly reduced Scc1 cleavage activity, although some residual activity remains [8,9,26]. The placement of the P1404L mutation also allowed us to PCR the C-terminus of *esp1-1* (including bp 4211) and use that fragment to integrate the mutation into the SGA starting strain as described [27].

The *esp1-1* query strain was mated to ∼4700 nonessential gene deletions (*geneXΔ)* [28] and ∼5300 strains from the *pGAL1/10-GST-ORFX* overexpression collection [29] to identify SL and SDL interactions, respectively. SL interactions were analyzed using a quantitative scoring program, with colony size functioning as a measure of growth in order to compare fitness of double mutants with *esp1-1* to the single deletion mutant [30]. We identified 161 double mutant strains that displayed a growth ratio below the determined cut-off for the screen (< 0.8 relative to the single deletion mutant control, p < 0.05) in 3/3 biological replicates, and consider these to be representative of SL interactions (S1 Table). Included in this list are two *esp1-1* SL interactors—*hsc82Δ* and *lte1Δ*- that have been previously described [18,31]. SDL genetic interactions were similarly examined by inducing *pGAL1/10-GST-ORFX* expression in both wild type and *esp1-1* mutants and comparing colony size. Plasmids were extracted from colonies identified in the screen, sequenced to confirm identity and retransformed into wild type and *esp1-1* mutant strains. Serial dilution assays were performed on dextrose versus galactose plates to confirm which genes caused a slow growth or lethal phenotype upon overexpression (S1 Fig). Ultimately, 44 different proteins were found to negatively impact the growth of the *esp1-1* mutant when overexpressed (S1 Fig, S2 Table). *SLK19*, a known proteolytic substrate of Esp1 that regulates spindle stability, was among the genes identified in the SDL screen, supporting the notion that increased dosage of a substrate in the presence of its defective enzyme can be detrimental to cells [15,29,32] (S1 Fig, S2 Table). There were only 2 genes identified in both the *esp1-1* SL and SDL screens, which is consistent with previous comparisons of SL and SDL screens [32–34]. Minimal overlap between the two screening methods is likely because SL and SDL screens probe different genetic interactions.

### Examination of confirmed negative *esp1-1* genetic interactions

**Fig 1:**
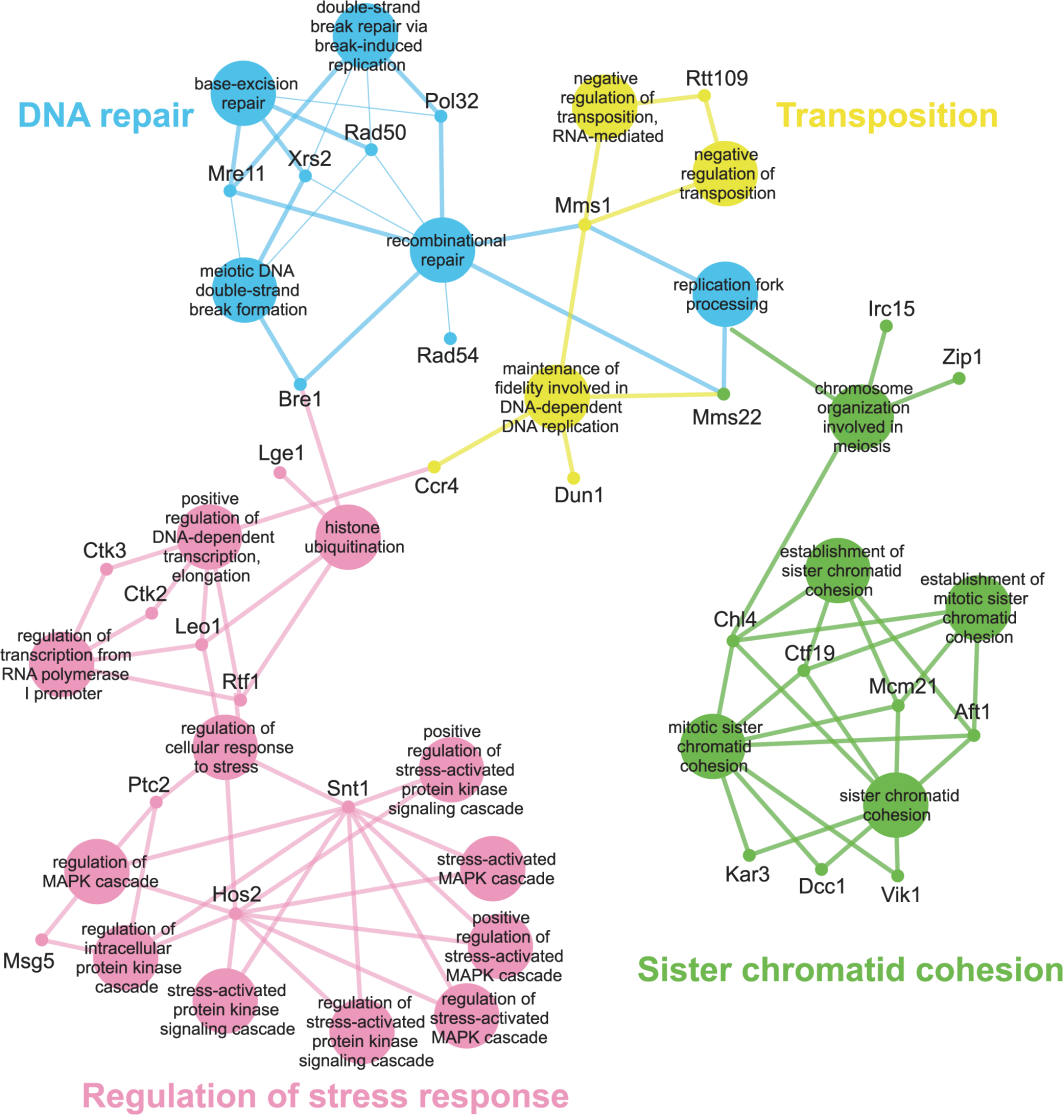
*esp1-1* functional interaction map derived from the SL SGA screen. The 161 genes identified in the *esp1-1* SL screen were analyzed using Cytoscape. All nodes represent significantly enriched (p <0.05) GO Terms in the dataset. Coloured nodes represent GO Terms that have been grouped into a category (written in the same colour) that is significantly enriched. Edges define associations between groups and edge thickness indicates the level of significance within the network. Genes identified in the SL screen that are associated with GO Terms are shown.

**Fig 2:**
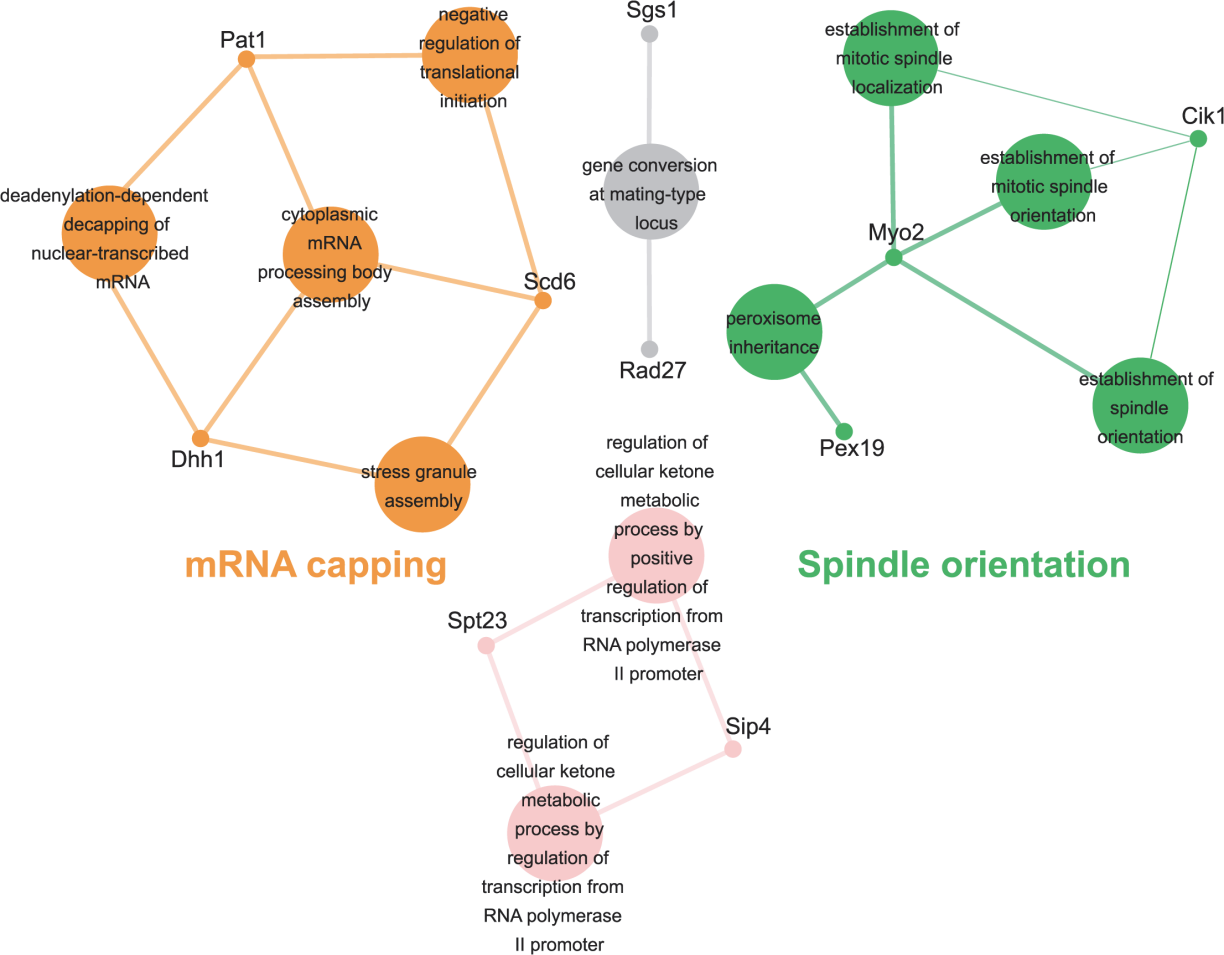
*esp1-1* functional interaction map derived from the SDL screen. The 44 genes identified in the *esp1-1* SDL screen were subjected to Cytoscape analysis. All nodes are significantly enriched (p <0.05) GO Terms in the dataset and coloured nodes represent GO Terms that have been grouped into a significantly enriched category. Grey nodes are GO Terms that were not grouped into an enriched category. Edges define associations between groups and edge thickness indicates the level of significance within the network. Genes identified in the SDL screen that are associated with GO Terms are shown.

Genes identified in the *esp1-1* SL screen were classified according to Gene Ontology (GO) Biological Process using Cytoscape to identify GO Terms that are enriched above genome frequency (p<0.05, S2 Fig) [35]. We also used the ClueGO plugin [36] to perform GO Term Grouping to distinguish which of these GO Terms correlated to a GO category that could be classified as significantly enriched (p<0.05, Fig. 1). As expected, Sister Chromatid Cohesion was among the enriched GO Term Groups. We also identified GO Terms that grouped into the category of DNA Repair which is consistent with a recently described role for Esp1 in promoting dissociation of cohesin during DNA double-strand break repair [37]. Notably, however, was the enrichment of Transposition and Regulation of Stress Response, two GO Term Groups that have no prior association to Esp1. We also examined the data from the *esp1-1* SDL screen in a similar manner and identified spindle orientation as an enriched GO Term Group, which is in agreement with the known role for Esp1 in spindle stability (Fig. 2, S2 Fig) [15,19,38]. In addition, we also identified mRNA capping as another GO Term Group that has no previous relation to Esp1 (Fig. 2, S2 Fig).

### *esp1-1* genetic interactors are involved in the life cycle of Ty1 retrotransposons

The retrotransposition cycle of Ty1 elements requires the following steps: (1) transcription of the *TY1* element by RNA polymerase II (2) nuclear export of the *TY1* mRNA (3) translation of *TY1* mRNA (4) assembly and maturation of Gag and Gag-Pol precursor proteins by Ty1-protease (5) reverse transcription of the *TY1* mRNA to cDNA (6) import to the nucleus and (7) integration of the cDNA into the genome [21]. We compared the 203 genes identified in our *esp1-1* SL and SDL screens to host *S*. *cerevisiae* genes required for individual steps of the Ty1 and Ty3 life cycle [39–43]. This analysis revealed that 48 (24%) of the *esp1-1* genetic interactors either promote or impede the Ty1 or Ty3 life cycle (Table 1). For example, genome-wide studies have implicated the P-body proteins Pat1, Dhh1 and Lsm1-7 in Ty1 and Ty3 retrotransposition as enhancers of the formation of retrotransposition-competent Ty virus like particles [40,41,44,45]. Pat1 and Dhh1 were identified in the *esp1-1* SDL screen whereas *lsm1Δ* and *lsm7Δ* were identified in the *esp1-1* SL screen (S1 and S2 Tables). The RecQ helicase Sgs1, which is synthetic dosage sensitive when overexpressed in *esp1-1* cells, is likely involved in the suppression of Ty1 cDNA recombination, as strains lacking *SGS1* transpose heterogeneous Ty1 multimers (S2 Table) [42,46]. The Fen-1 nuclease Rad27, which is also SDS when overexpressed in *esp1-1* cells, is thought to degrade Ty1 cDNA to prevent the formation of Ty1 multimers (S2 Table) [42,47]. Further, members of the Paf1 complex serve to inhibit Ty1 transposition, possibly post-transcriptionally or by limiting the amount of favourable hotspots for integration [42]. Deletion mutants of three members of the Paf1 complex—*cdc73Δ*, *leo1Δ* and *rtf1Δ* - caused an SL phenotype when combined with *esp1-1* (S1 Table). Two *esp1-1* SDL genes—*SPT10* and *SPT23*—are named because of their identification in “suppressors of Ty” screens (S2 Table) [48,49]. Either mutation (*SPT10)* or multicopy expression (*SPT23*) of these genes confers suppression of Ty1-induced promoter insertion mutations [48,49]. Clearly, several genetic interactors of *esp1-1* have important roles in promoting or restricting Ty1 transposition.

**Table 1:** Genes identified in *esp1-1* SL and SDL screens implicated in Ty1 and Ty3 transposition.

ORF	Gene	*esp1-1* screen	Ty1	Ty3
*YIL040W*	*APQ12* ^*a*^	SL	Promote	
*YNL242W*	*ATG2* ^*k*^	SL		Inhibit
*YBR068C*	*BAP2* ^*a*^	SL	Promote	
*YDL074C*	*BRE1* ^*b*^ ^,^ ^*k*^	SL	Inhibit	Inhibit
*YAL021C*	*CCR4* ^*a*^	SL	Promote	
*YPR119W*	*CLB2* ^*k*^	SL		Inhibit
*YJL006C*	*CTK2* ^*k*^	SL		Promote
*YCL016C*	*DCC1* ^*c*^	SL	Promote	
*YFL001W*	*DEG1* ^*k*^	SL		Promote
*YDL160C*	*DHH1* ^*a*^ ^,^ ^*d*^ ^,^ ^*k*^	SDL	Promote	Promote
*YBR269C*	*FMP21* ^*k*^	SL		Inhibit
*YOR202W*	*HIS3* ^*a*^	SL	Promote	
*YGL194C*	*HOS2* ^*e*^	SL	Promote	
*YDL115C*	*IWR1* ^*c*^	SL	Promote	
*YPL125W*	*KAP120* ^*k*^	SL		Promote
*YOR123C*	*LEO1* ^*b*^	SL	Inhibit	
*YPL055C*	*LGE1* ^*a*^	SL	Promote	
*YJL124C*	*LSM1* ^*a*^ ^,^ ^*c*^ ^,^ ^*d*^	SL	Promote	
*YPR164W*	*MMS1* ^*b*^	SL	Inhibit	
*YLR320W*	*MMS22* ^*a*^ ^,^ ^*b*^ ^,^ ^*c*^	SL	Promote/Inhibit	
*YMR224C*	*MRE11* ^*b*^ ^,^ ^*f*^	SL	Inhibit	
*YGL221C*	*NIF3* ^*a*^	SL	Promote	
*YPR164W*	*NUT2* ^*f*^	SL	Inhibit	
*YCR077C*	*PAT1* ^*c*^ ^,^ ^*d*^	SDL	Promote	
*YJR043C*	*POL32* ^*a*^	SL	Promote	
*YKL113C*	*RAD27* ^*b*^	SDL	Inhibit	
*YNL250W*	*RAD5* ^*b*^ ^,^ ^*f*^ ^,^ ^*g*^	SL	Inhibit	
*YGL163C*	*RAD54* ^*b*^ ^,^ ^*g*^	SL	Inhibit	
*YDR156W*	*RPA14* ^*b*^	SL	Inhibit	
*YNL069C*	*RPL16B* ^*a*^ ^,^ ^*c*^	SL	Promote	
*YHR010W*	*RPL27A* ^*a*^	SL	Promote	
*YFL036W*	*RPO41* ^*a*^	SL	Promote	
*YHR021C*	*RPS27B* ^*a*^	SL	Promote	
*YGL244W*	*RTF1* ^*b*^ ^,^ ^*c*^	SL	Promote/Inhibit	
*YLL002W*	*RTT109* ^*b*^ ^,^ ^*f*^ ^,^ ^*k*^	SL	Inhibit	Inhibit
*YMR190C*	*SGS1* ^*b*^ ^,^ ^*f*^ ^,^ ^*k*^	SDL	Inhibit	Inhibit
*YJL089W*	*SIP4* ^*a*^	SDL	Promote	
*YGR229C*	*SMI1* ^*c*^	SL	Promote	
*YCR033W*	*SNT1* ^*a*^	SL	Promote	
*YJL127C*	*SPT10* ^*a*^ ^,^ ^*c*^ ^,^ ^*h*^	SDL	Promote	
*YKL020C*	*SPT23* ^*a*^ ^,^ ^*i*^	SDL	Promote	
*YOR212W*	*STE4* ^*j*^	SDL	Inhibit	
*YPL157W*	*TGS1* ^*a*^	SL	Promote	
*YPR133W-A*	*TOM5* ^*a*^	SL	Promote	
*YNL299W*	*TRF5* ^*a*^	SL	Promote	
*YDR200C*	*VPS64* ^*b*^	SL	Inhibit	
*YDR369C*	*XRS2* ^*c*^	SL	Promote	
*YDR290W*	*YDR290W* ^*k*^	SL		Inhibit

^a^Mutants that suppress the hypertransposition phenotype of *rtt101Δ* and *med1Δ* mutants [45]

^b^Mutants that increase the Ty1 transposition frequency [42]

^c^All mutants have decreased Ty1 transposition except for 2 (*YML105C* and *YOL159C*) [40]

^d^P-body components encoded by *DHH1*, *KEM1*, *LSM1*, and *PAT1* act as cofactors that posttranscriptionally enhance Ty1 retrotransposition [44]

^e^Deletion of *HOS2* or *SET3* reduces Ty1 transposition [101]

^f^ *rtt* mutants have increased levels of Ty1 transposition [43]

^g^ *rad50*, *rad51*, *rad52*, *rad54* and *rad57* mutants have increased Ty1 transposition [58]

^h^ *spt* mutants suppress *lys2-128δ* and *his4-917* Ty insertion mutations [49]

^i^ *SPT23* is a high copy suppressor of the *his4-912δ* and *lys2-61* Ty induced Promoter mutations [48]

^j^The mating pheromone-response pathways suppresses Ty1 transposition[102]

^k^Mutants that increase or decrease Ty3 transposition frequency [41]

### Mass spectrometry reveals a physical interaction for Esp1 with Ty1-IN

**Fig 3:**
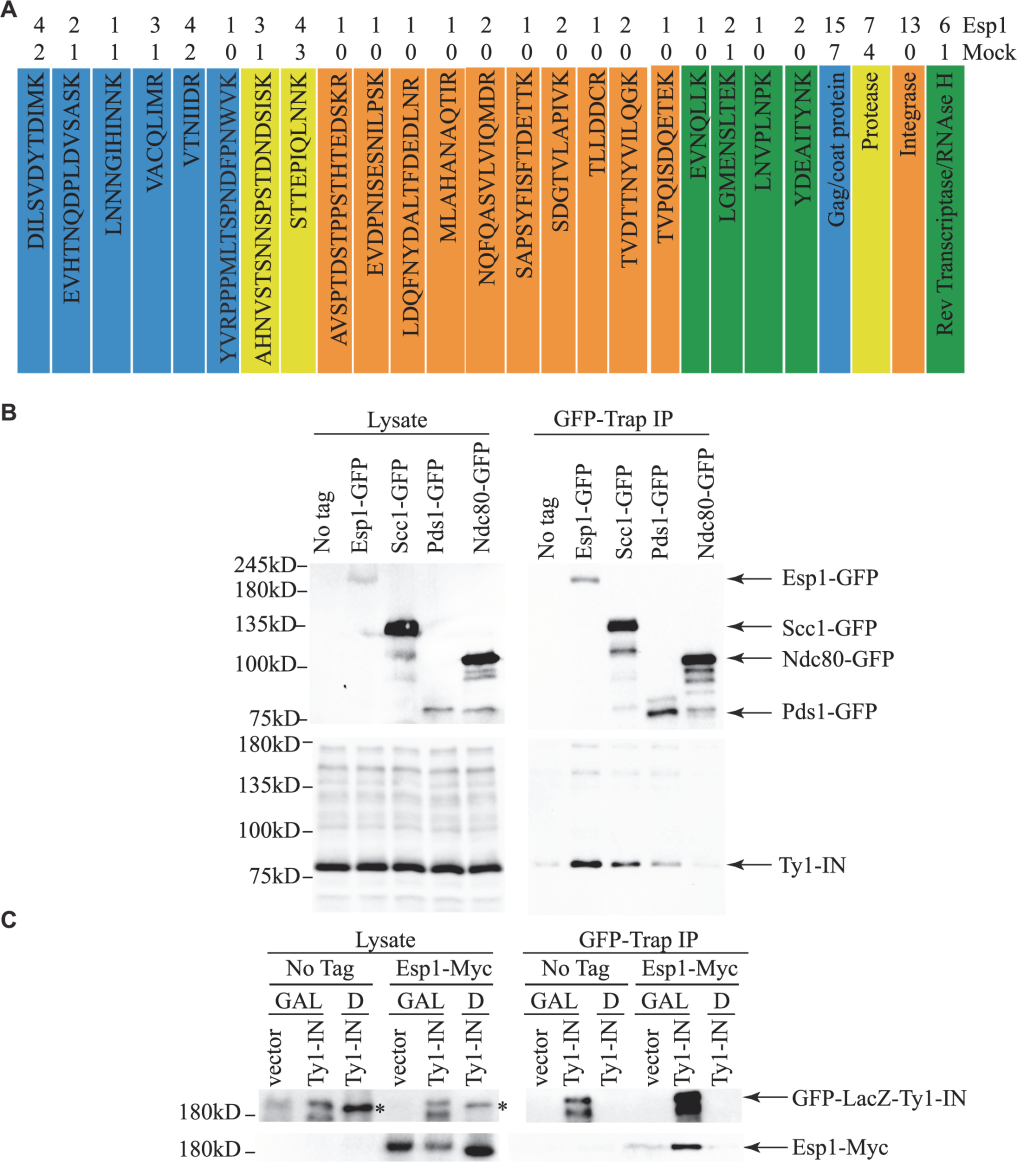
Esp1 physically interacts with Ty1-IN. (A) Ty1 peptides identified in Esp1-Myc mass spectrometry versus untagged (mock) strain are color coded as follows: Gag/coat protein (blue), PR (yellow), IN (orange) and RT/RNAse H (green). (B) Immunoblot of whole cell lysate (Lysate) and GFP-Trap IP carried out from untagged wild type (No Tag), Esp1-GFP, Scc1-GFP, Pds1-GFP and Ndc80-GFP cells. Expression of a Ty1 element (*pGAL1-Ty1-H3*) was induced in all strains for 24 hours prior to cell lysis. Blots were probed with anti-GFP and anti-IN (8b11) antibodies. (C) Immunoblot of whole cell lysate (Lysate) and GFP-Trap IP carried out from Esp1-Myc or untagged wild type (No Tag) cells carrying a pGAL-GFP-LacZ-Ty1-IN plasmid (Ty1-IN) or pGAL-GFP-lacZ (vector) control. Cells were either grown in glucose (D) or galactose (GAL) for 24 hours to repress or induce GFP-LacZ-Ty1-IN expression, respectively. The asterisk marks a background band that is present in the lysate of the cells grown in glucose but is not detected in the GFP-Trap IP.

To corroborate our genetic interaction data, and better understand its functional significance, a search for Esp1 physical interacting proteins was also undertaken. We performed an immunoprecipitation (IP) of 13Myc-tagged Esp1 from logarithmically growing cells, followed by mass spectrometry analysis that we compared to a mock IP performed in parallel with an untagged strain. More specifically, we compared the total number of peptides for each protein identified by mass spectrometry (i.e., spectral counting), and we identified 152 proteins that co-purified with Esp1 but were not enriched in the mock experiment (S3 Table). The identification of the separase inhibitor, Pds1/securin—with 16 peptides identified in the Esp1 IP and none in the mock—served as an internal experimental control. The Esp1 co-purified proteins included 11 proteins implicated in Ty1 transposition such as the “suppressors of Ty” Spt6 and Spt16 (Table 2) [50–52]. Notably, we also identified 13 peptides corresponding to Ty1-IN while none were identified in the mock IP and observed a six-fold increase of spectral counts for Ty1 peptides corresponding to the Ty1-RT (Fig. 3A). In comparison, peptides from the Ty1-Gag and Ty1-PR were readily identified in both the mock and Esp1 IPs, suggesting these Ty1 proteins may not specifically interact with Esp1. A potential interaction between Esp1 and Ty1-IN suggests that Esp1 may have a direct role in Ty1 element transposition.

**Table 2:** Genes identified in Esp1-13Myc mass spectrometry screen implicated in Ty1 transposition.

ORF	Name	Ty1Transposition
*YLR384C*	*IKI3* ^*a*^	Promote
*YNL307C*	*MCK1* ^*a*^	Promote
*YKL009W*	*MRT4* ^*b*^	Promote
*YMR080C*	*NAM7* ^*b*^	Promote
*YER070W*	*RNR1* ^*c*^	Inhibit
*YGR229C*	*SMI1* ^*a*^	Promote
*YEL031W*	*SPF1* ^*b*^	Promote
*YGL207W*	*SPT16* ^*d*^	Promote
*YGR116W*	*SPT6* ^*e*^	Inhibit
*YMR125W*	*STO1* ^*a*^	Promote
*YGR285C*	*ZUO1* ^*b*^	Promote

^a^All mutants have decreased Ty1 transposition except for *YML105C* and *YOL159C* [40]

^b^Mutants that suppress the hypertransposition phenotype of *rtt101Δ* and *med1Δ* mutants [45]

^c^ *rtt* mutants have increased levels of Ty1 transposition [43]

^d^ *SPT16* is a high-copy suppressor of Ty1-δ insertion mutations [50,51]

^e^ *spt* mutants suppress *his4-912δ* Ty insertion mutations [52]

We validated the physical interaction of Esp1 with Ty1 proteins by co-IP experiments using a different Esp1 epitope tag (GFP) to demonstrate specificity of the interaction. Although the Ty1-Gag protein can be detected at endogenous levels, the TyB polypeptides cannot using available reagents and must be overexpressed [53]. Therefore, *pGAL1Ty1-H3*, a Ty1 expression plasmid, was induced in both an untagged control strain and Esp1-GFP strain. Esp1-GFP was purified using GFP-Trap beads, immunoblot analysis performed and probed for the presence of Ty1-IN. We found that Ty1-IN was specifically present in the Esp1-GFP IP but not in the purification from the untagged strain (Fig. 3B). We could only detect processed Ty1-IN in the lower molecular weight region of the co-IP and not in the region where unprocessed PR-IN or IN-RT polypeptides would migrate (Fig. 3B). Similarly, we did not detect any peptides in our MS data that spanned the unprocessed PR-IN or IN-RT polypeptides. However, we cannot completely exclude the possibility that Esp1 also binds, to a lower extent, to the unprocessed or partially cleaved TyB polypeptide.

Esp1 interacts with Pds1/securin immediately after *PDS1* expression in S phase and remains bound until Pds1 is targeted for degradation by the Anaphase Promoting Complex, whereby Esp1 is released to cleave the Scc1 cohesin [8,9,38]. We were therefore interested to test whether Pds1 and Scc1 could also physically interact with Ty1-IN. GFP-tagged Pds1 and Scc1 were purified individually from yeast lysates and immunoblot analysis performed. In comparison to Esp1, the interaction of Pds1-GFP with Ty1-IN was much weaker and just above background levels (Fig. 3B). We found that Scc1-GFP interacted with Ty1-IN. However, in comparison to Esp1-GFP, less Ty1-IN was detected in the co-IP despite higher expression of Scc1-GFP (Fig. 3B). Therefore, Ty1-IN interacts predominantly with Esp1 and to a lesser extent, Scc1. Ty1-IN does not interact with the Ndc80-GFP kinetochore protein demonstrating that GFP fusions in general do not interact with Ty1-IN (Fig. 3B). To further validate the Esp1-Ty1-IN interaction, we purified an overexpressed version of Ty1-IN fused to GFP-lacZ (pGAL-GFP-LacZ-Ty1-IN) using GFP Trap beads and tested if GFP-LacZ-Ty1-IN interacted with 13Myc tagged Esp1. Esp1-Myc was present in the purification of GFP-LacZ-Ty1-IN but not in a purification where GFP-LacZ-Ty1-IN was not expressed [pGFP-lacZ-Ty1-IN Dextrose (D)] or in a purification with vector control (Fig. 3C). In summary, we have demonstrated using mass spectrometry and two independent co-IP experiments that Esp1 interacts with Ty1-IN.

**Fig 4:**
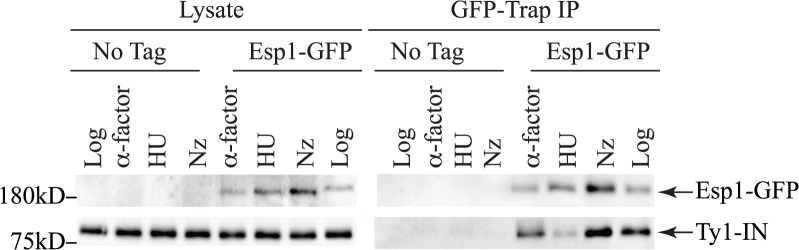
Esp1 interacts with Ty1-IN in G1 and G2/M phase cells. Untagged (No Tag) and Esp1-GFP tagged cells carrying the *pGAL1-Ty1-H3* element were induced for 24 hours with 2% galactose, then arrested in G1 phase with mating pheromone (α-factor), S phase with hydroxyurea (HU) and G2/M phase with Nocodazole (Nz). Immunoblot of whole cell lysate (Lysate) and GFP-Trap IP from Log, α-factor, HU and Nz arrested cells is shown. Blots were probed with anti-GFP and anti-IN (8b11) antibodies.

Esp1 is expressed throughout the cell cycle with about three-fold lower levels in G1 phase than the rest of the cell cycle [38]. We queried if Esp1 interacts with Ty1-IN at a particular stage of the cell cycle. After induction of Ty1 expression in Esp1-GFP cells for 24 hours, we arrested cells in G1 phase with mating pheromone (α-factor), S phase with hydroxyurea (HU) and G2 phase with nocodazole (Nz) (Fig. 4). Esp1-GFP was purified with GFP-Trap beads and immunoblot analysis performed to test for interaction with Ty1-IN. We detected an interaction between Esp1-GFP and Ty1-IN in logarithmically growing, α-factor and Nz arrested cells, but a minimal interaction was detected in HU arrested cells (Fig. 4).

**Fig 5:**
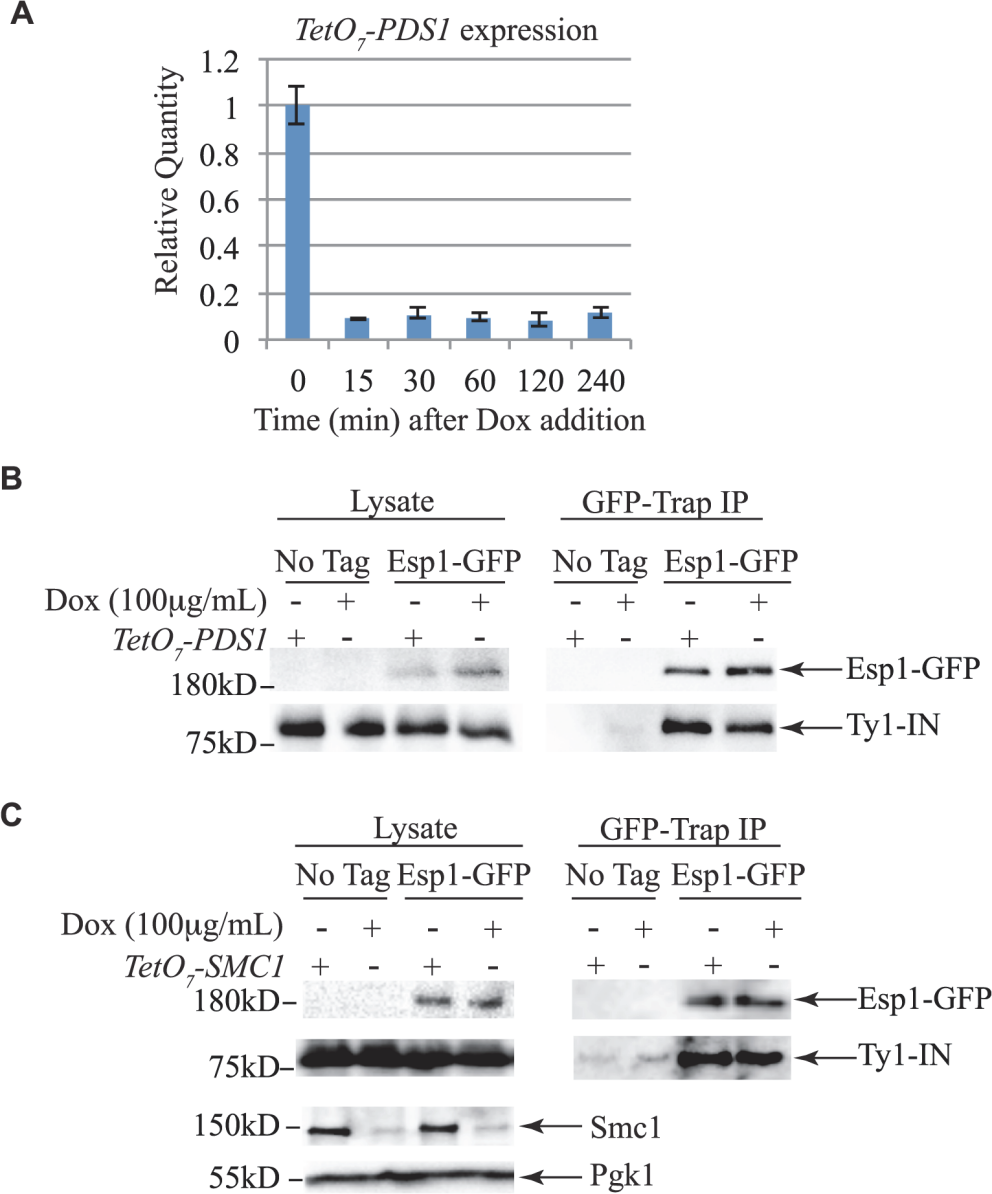
Pds1 and Smc1 are not required for the Esp1-Ty1-IN interaction. (A) Quantitative PCR analysis of *PDS1* expression driven from the *TetO*_*7*_ promoter after 100μg/mL doxycycline addition. The relative quantity of *PDS1* transcript compared to a control (*TAF10*) is shown. (B) Esp1-GFP and untagged (No Tag) cells containing the *TetO*_*7*_-*PDS1* allele and *pGAL1-Ty1-H3* were induced for 24 hours with 2% galactose, then 100μg/mL doxycycline was added (+) or not (-) for one hour. Esp1-GFP was purified with GFP-Trap beads, immunoblot analysis performed and probed with anti-GFP and anti-IN (8b11) antibodies. (C) Esp1-GFP and untagged (No Tag) cells containing the *TetO*_*7*_—*SMC1* allele and *pGAL1-Ty1-H3* were induced for 24 hours with 2% galactose, then 100μg/mL doxycycline was added (+) or not (-) for six hours. Esp1-GFP was purified as in (B). The depletion of Smc1 was monitored with anti-Smc1 antibodies and the lysate was probed with anti-Pgk1 as a loading control.

Pds1 is expressed in G1/S phase and degraded at the metaphase to anaphase transition [5,54]. To determine if Pds1 is required for Esp1 to interact with Ty1-IN, we used a strain in which Pds1 expression is controlled by a tetracycline (*TetO*_*7*_)-regulatable promoter that is inhibited in the presence of doxycycline (Dox) [55]. Dox addition to *TetO*_*7*_ *-PDS1* cells inhibited *PDS1* expression after 15min of treatment and *PDS1* transcript levels were reduced for up to 240min (Fig. 5A). Since Pds1 is degraded every cell cycle, we treated TetO_7_ *-PDS1* cells for one hour with Dox to ensure that all Pds1 protein was removed from the cell [5]. Esp1-GFP was purified from Dox treated (+) and untreated (-) *TetO*_*7*_ *-PDS1* cells and was able to interact with Ty1-IN in the presence or absence of Pds1 (Fig. 5B). We performed a similar experiment with a *TetO*_*7*_ *-SMC1* strain to determine if cohesin is required for the Esp1-Ty1-IN interaction. We first determined that treatment of the *TetO*_*7*_ *-SMC1* strain with Dox for 6 hours was sufficient to reduce Smc1 to background levels, as determined by immunoblot with an anti-Smc1 antibody (Fig. 5C, Smc1 panel). We found that, similar to Pds1, when Smc1 was depleted by Dox treatment, Esp1-GFP was still able to interact with Ty1-IN (Fig. 5C). Our results strongly indicate that the interaction of Esp1 with Ty1-IN does not depend on Pds1 or Smc1.

### *esp1* and *pds1* mutants have defects in Ty1 transposition whereas cohesin mutants have increased Ty1 transposition

**Fig 6:**
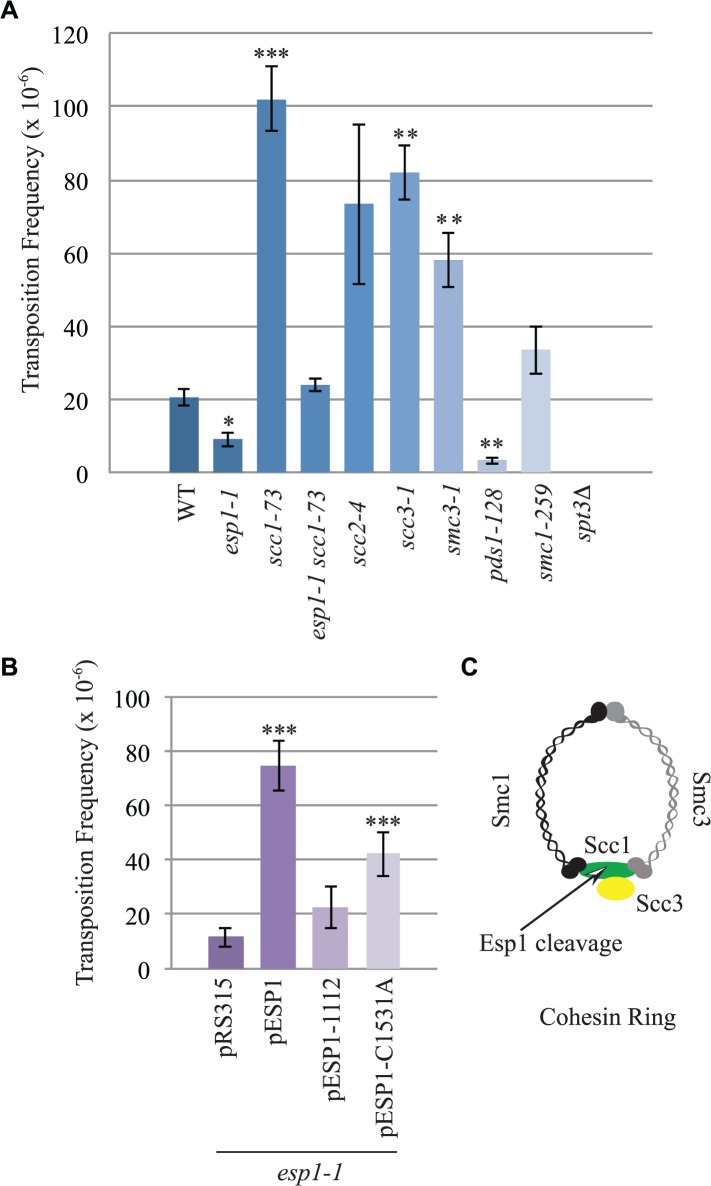
*esp1* and *pds1* mutants have decreased, whereas cohesin mutant have increased Ty1 mobility. (A) Transposition frequency of wild type (WT), *esp1-1*, *scc1-73*, *esp1-1 scc1-73*, *scc2-4*, *scc3-1*, *smc3-1*, *pds1-128*, *smc1-259*, and *spt3Δ* (control) cells carrying a *CEN* plasmid with a marked Ty1element expressed from its endogenous promoter (pBDG922). (B) Transposition frequency of the *esp1-1* mutant carrying either a vector (pRS315), wild type Esp1 (*pESP1*), an Esp1 protease domain truncation (*pESP1-1112*) or an Esp1 catalytic site mutation (*pESP1-C1531A*) as well as pBDG922. In both (A) and (B), cells were induced to transpose for 4 days at 20°C. A minimum of three independent transformants were quantified. Mutants with transposition frequencies that are significantly different from wild type (A) or pRS315 (B) are shown with one (p<0.05), two (p<0.005) or three asterisks (p<0.0005). (C) Structure of the cohesin ring.

The interaction of Esp1 with Ty1-IN suggests that Esp1 may be required for Ty1 transposition. We performed a quantitative assay for Ty1 mobility with a plasmid (pBDG922) carrying a Ty1 element expressed from its endogenous promoter. The Ty1 element contains a *HIS3* gene with an artificial intron (AI) interrupting its expression. After Ty1 expression, the intron is removed from the *Ty1-H3mHIS3AI* mRNA intermediate, and successful Ty1 cDNA integration renders the strain *HIS3+*[56]. Wild type, *esp1-1* and other mutant strains carrying the pBDG922 plasmid were induced to transpose at 20°C for 4 days before plating onto selective media to measure both viability and Ty1 transposition. Spt3 is required for transcription of Ty1 elements, therefore the *spt3* deletion mutant is included as a negative control [57]. Wild type cells underwent Ty1 transposition at a frequency of 20.8 x 10^–6^ compared to 9.2 X 10^–6^ for *esp1-1* cells (∼2-fold less than wild type, p < 0.05, Fig. 6A). We also found that *pds1-128* cells had significantly reduced Ty1 transposition frequency at 3.5 x10^-6^ (∼6-fold less than wild type, p<0.005). We tested a panel of cohesin mutants including the Scc1 cohesin that is cleaved by Esp1 (Fig. 6C). There was a 5-fold increase in the Ty1 transposition frequency in the *scc-173* mutant (Fig. 6A, p<0.0005), which suggested that the presence of cohesin might impede Ty1 transposition. Indeed, strains carrying mutations in the Scc3 and Smc3 cohesin proteins also displayed significantly increased Ty1 transposition frequency (Fig. 6A). We were unable to determine if mutation of the cohesin loader protein (*scc2-4*) also results in a high transposition frequency because of variability amongst the replicate samples. We tested if the *esp1-1* mutant may be deficient in Ty1 transposition because of the inability to properly cleave Scc1. We found that the Ty1 transposition frequency was restored to wild type levels in the *esp1-1 scc1-73* double mutant (Fig. 6A) suggesting that removal of cohesin can restore transposition to *esp1-1* cells.

The increase in transposition frequency that we detected in the cohesin mutants suggests that removal of cohesin by the Esp1 protease may be necessary for transposition to occur. Since a strain carrying a mutation in the catalytic domain of Esp1 is not viable, we tested if expressing a catalytically dead version of Esp1 was able to rescue the transposition defect of an *esp1-1* strain, compared to expression of wild type Esp1. We first demonstrated that expression of wild type *ESP1* (*pESP1*) from a plasmid was capable of restoring the transposition frequency of an *esp1-1* mutant carrying vector alone (pRS315) from 11.6x10^-6^ to 74.7 x 10^–6^, a 6.4-fold increase (p<0.0005, Fig. 6B). When we expressed *pESP1-C1531A*, which carries a cysteine to alanine mutation at amino acid residue 1531 in the catalytic domain of Esp1, the transposition frequency was partially restored to 42.2x 10^–6^, a 3.6-fold increase compared to *esp1-1* alone (p<0.005, Fig. 6B). Since we were able to restore approximately half of the Esp1 transposition activity with the catalytically inactive version of Esp1, the catalytic activity may only be partially required for transposition. To determine if the Esp1 protease domain is required for transposition, we removed the C-terminal 518 amino acids that comprise the protease domain (pESP1-1112). The transposition frequency in the *pESP1-1112* mutant was not significantly different than the *esp1-1* mutant carrying vector alone suggesting that the protease domain is required for Ty1 transposition.

### Ty1 cDNA levels are not altered in *esp1* and *pds1* mutants and Ty1 polypeptide processing does not require separase

**Fig 7:**
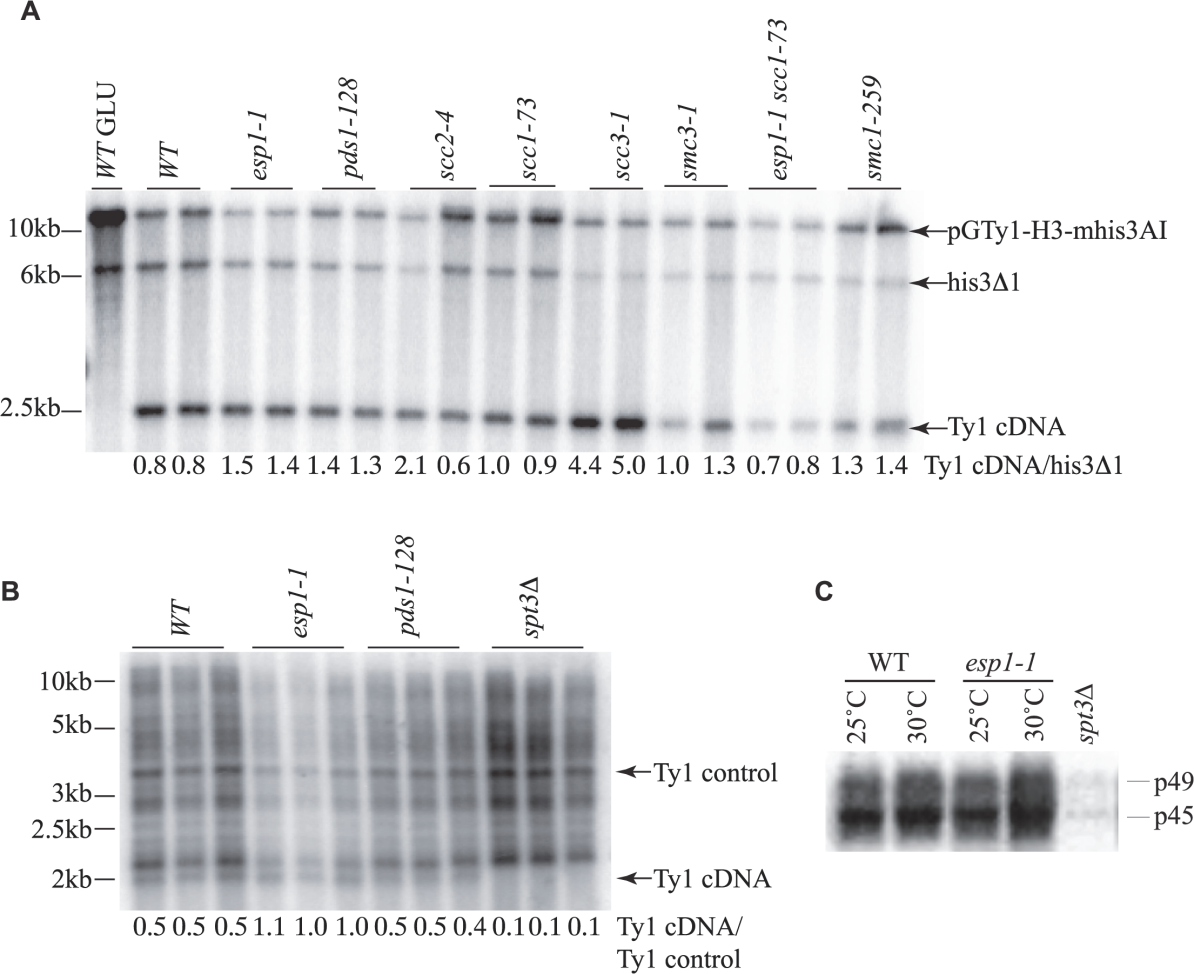
Ty1 cDNA levels are not affected in *esp1* and *pds1* mutants. (A) Southern blot analysis of *AflII* digested yeast genomic DNA isolated from the indicated mutants carrying the *pGTy1-H3-mhis3AI* plasmid after 24 hour induction with 2% galactose. One wild type (WT) sample was also grown in 2% glucose (GLU) as a control for *TY1* element expression. The blot was probed with a radiolabeled *HIS3* gene. Shown are the fragment sizes for Ty1 cDNA (∼2.4kb), the endogenous *HIS3* locus of the S288C deletion strain with 200bp deleted (*his3Δ1*, ∼6.5kb) and the *pGTy1-H3-mhis3AI* plasmid which is linearized (∼14kb). Below each lane is the quantitative ratio of Ty1 cDNA signal to *his3Δ1* signal. (B) Southern blot analysis of *PvuII* digested yeast genomic DNA isolated from the indicated mutants after 2 days growth at 20°C. Endogenous Ty1 elements and cDNA were detected with a radiolabelled *PvuII/SnaBI* Ty1 element fragment. Below each lane is the quantitative ratio of the Ty1 endogenous cDNA (∼2kb) to an endogenous Ty element (Ty1 control). *spt3Δ* serves as a negative control for Ty1 cDNA levels. (C) Endogenous Ty1-Gag processing in wild type (WT) versus *esp1-1* cells grown at 25°C or incubated for 6 hours at 30°C assessed by immunoblot. Blot was probed with anti-Gag antibody. p49 = unprocessed Gag; p45 = processed Gag. *spt3Δ* serves as a negative control for Gag expression.

The reduction of Ty1 transposition in *esp1* and *pds1* mutants could be due to defects at various stages of the Ty1 life cycle. The conversion of Ty1 RNA to cDNA, which occurs mid-way through the Ty1 life cycle, is considered a rate-limiting step in Ty1 transposition and mutants with increased or decreased Ty1 cDNA levels typically display an increased or decreased Ty1 transposition frequency, respectively [40,58]. To determine if Ty1 cDNA production is affected in *esp1*, *pds1* or cohesin mutants we first used a Ty1 expression plasmid similar to that used for the transposition assays, except this Ty1 element is under control of a *GAL1* promoter to facilitate visualization of the Ty1 cDNA by southern blot analysis. A probe spanning the *HIS3* gene was used to detect Ty1 cDNA, the native *his3Δ1* locus and the plasmid as described (Fig. 7A, [40]). We quantitated the Ty1 cDNA relative to the *his3Δ1* locus, which gave a ratio of 0.8 for both wild type isolates (Fig. 7A). The Ty1 cDNA ratio was slightly higher for both *esp1-1* and *pds1-128* strains suggesting that the defect in Ty1 transposition in these mutants is not due to lower Ty1 cDNA production. The ∼5-fold increase in Ty1 transposition detected in the *scc1-73* cohesin mutant (Fig. 6A), cannot be accounted for by the Ty1 cDNA ratio which is similar to wild type levels (1.0 and 0.9 for *scc1-73* compared to 0.8 for wild type, Fig. 7A). Likewise the *smc3-1* cohesin mutant had Ty1 cDNA ratios of 1.0 and 1.3 but a ∼3-fold increase in Ty1 transposition frequency (Fig. 6A, 7A). The *scc3-1* mutant however, which displayed a ∼4-fold increase in Ty1 transposition (Fig. 6A) had significantly increased Ty1 cDNA levels compared to the *his3Δ1* locus which could account for increased transposition (Fig. 7A).

The transposition defects that we detected in *esp1-1* and *pds1-128* mutants were based on Ty1 expression from a plasmid with a native Ty1 promoter (Fig. 6A). Therefore we also analyzed Ty1 cDNA endogenous levels to confirm that *esp1-1* and *pds1-128* mutants had no defects in production of endogenous Ty1 cDNA (Fig. 7B). In this case, yeast genomic DNA was digested with *PvuII* which releases a linear ∼2kb fragment of Ty1 cDNA [59]. The southern blot was probed with a C-terminal fragment of the Ty1 element that detects both the endogenous Ty1 cDNA and Ty1 elements. We quantitated the ratio of Ty1 cDNA to a Ty1 element fragment detected by the probe (numbers below each lane on the blot). Our analysis confirmed that *esp1-1* and *pds1-128* mutant have similar levels of Ty1 cDNA to wild type cells (Fig. 7B).

While the protease activity of Esp1 was at least partially dispensable for transposition, we verified that Esp1 did not affect the processing of the TyA-TyB polypeptide. Accordingly, the ratio of endogenous Ty1 Gag precursor and mature protein levels were not appreciably different in an *esp1-1* strain, suggesting that it is unlikely that the TyA-TyB polypeptide is subject to separase-mediated proteolysis (Fig. 7C).

### Ty1 element insertion events are reduced in *esp1* and *pds1* mutants but increased in cohesin mutants

**Fig 8:**
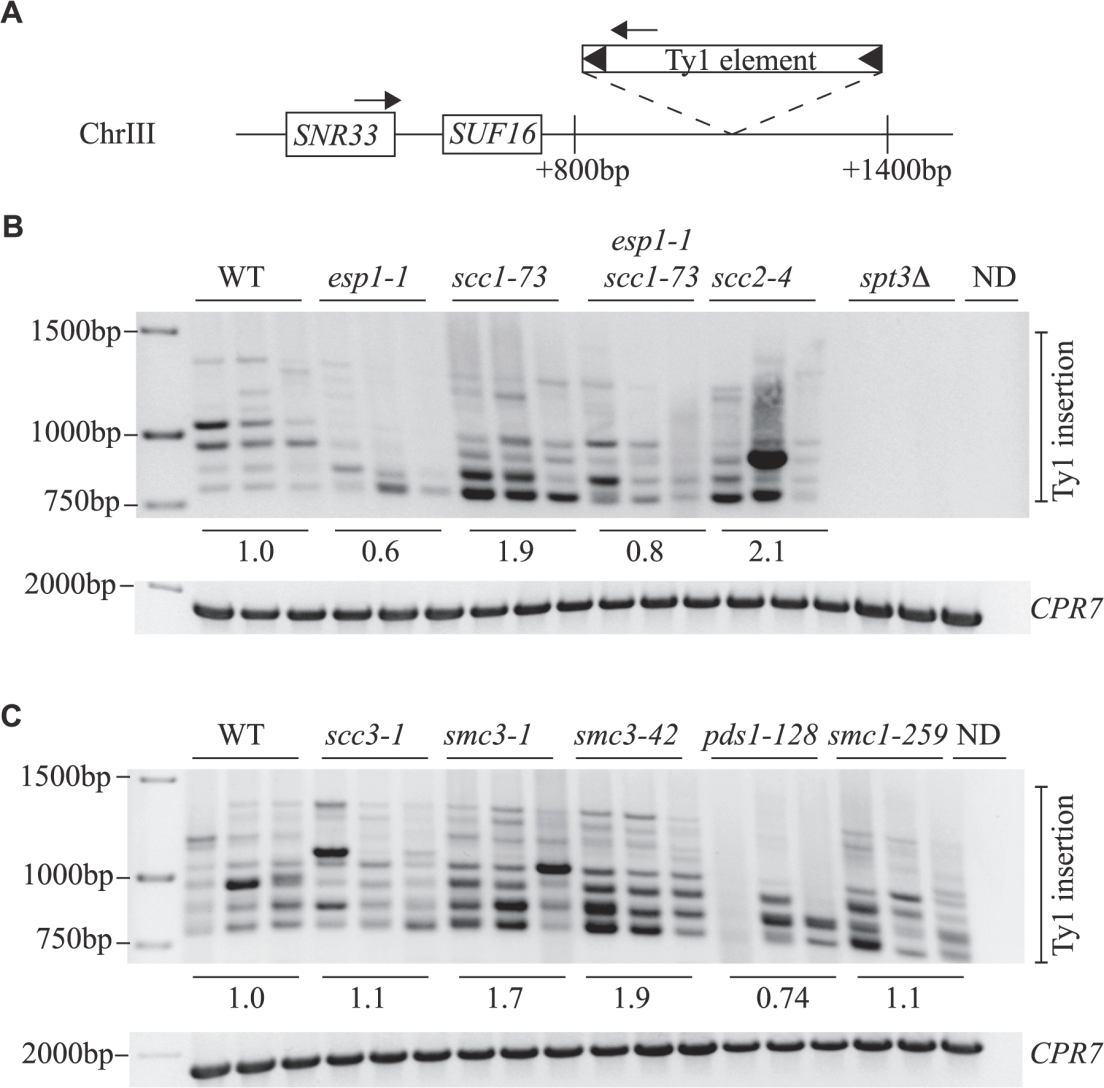
Esp1 and Pds1 are required for Ty1 element insertion upstream of the *SUF16* locus. (A) Schematic of the *SUF16* genomic locus with PCR primers (arrows) designed to hybridize within the *SNR33* gene and the newly inserted Ty1 elements. (B) and (C) PCR analysis of yeast genomic DNA extracted from indicated strains grown in triplicate for 3 days at 20°C to induce transposition. Upper panel is the result of the PCR assay with primers shown in (A) whereas lower panel is a control PCR for the *CPR7* locus to demonstrate that yeast genomic DNA was present in each sample. Quantification of Ty1 insertion events is shown relative to wild type (WT) as described in the Materials and Methods.

The *SUF16* glycine tRNA gene locus is a hotspot for Ty1 transposition [60]. We induced endogenous Ty1 transposition by growing wild type and mutant cells at 20°C for 3 days, isolated genomic DNA, then measured *de novo* transposition upstream of the *SUF16* locus using an established PCR assay [42]. One primer hybridizes with the *SNR33* locus adjacent to *SUF16* instead of *SUF16* because of the repetitive nature of tRNA genes. The second primer hybridizes within the Ty1 element [42]. At this particular hotspot, Ty1 element insertions occur in a window of ∼800bp to 1500bp upstream of *SUF16* in a periodic fashion due to nucleosome positioning [61,62]. Although Ty1 elements can insert in either orientation, this PCR analysis only detects Ty1 elements transcribed towards the *SUF16* locus (Fig. 8A). Each strain was grown in triplicate and panels B and C represent genomic DNA isolated from two separate experiments. *spt3Δ* is included as a control for a strain that does not undergo transposition (Fig. 8B). For quantification, we summed the intensity of Ty1 insertions for each strain and compared the value to wild type. *esp1-1* and *pds1-128* mutants have less Ty1 insertion events compared to the wild type strains—60% and 74% that of wild type, respectively (Fig. 8B, C). This data correlates well with the reduced frequency of transposition in *esp1-1* and *pds1-128* mutants (Fig. 6A). The *scc1-73* and *smc3-1* and *smc3-42* cohesin mutants have increased Ty1 insertion events compared to wild type cells (1.9, 1.7 and 1.9-fold higher than wild type, respectively, Fig. 8B, C). Both *scc1-73* and *smc3-1* mutants displayed increased transposition frequency in our quantitative assay (*smc3-42* was not tested) suggesting that cohesin mutants allow increased Ty1 insertion events (Fig. 6A, 8B, C). The *scc3-1* mutant however had increased Ty1 transposition frequency but not Ty1 insertion events upstream of the *SUF16* locus (Fig. 6A, 8C). Overall, our data confirms that removal of cohesin is beneficial for Ty1 transposition and that both Esp1 and Pds1 are required for efficient Ty1 transposition.

### The entire Esp1 polypeptide contributes to Ty1 transposition

**Fig 9:**
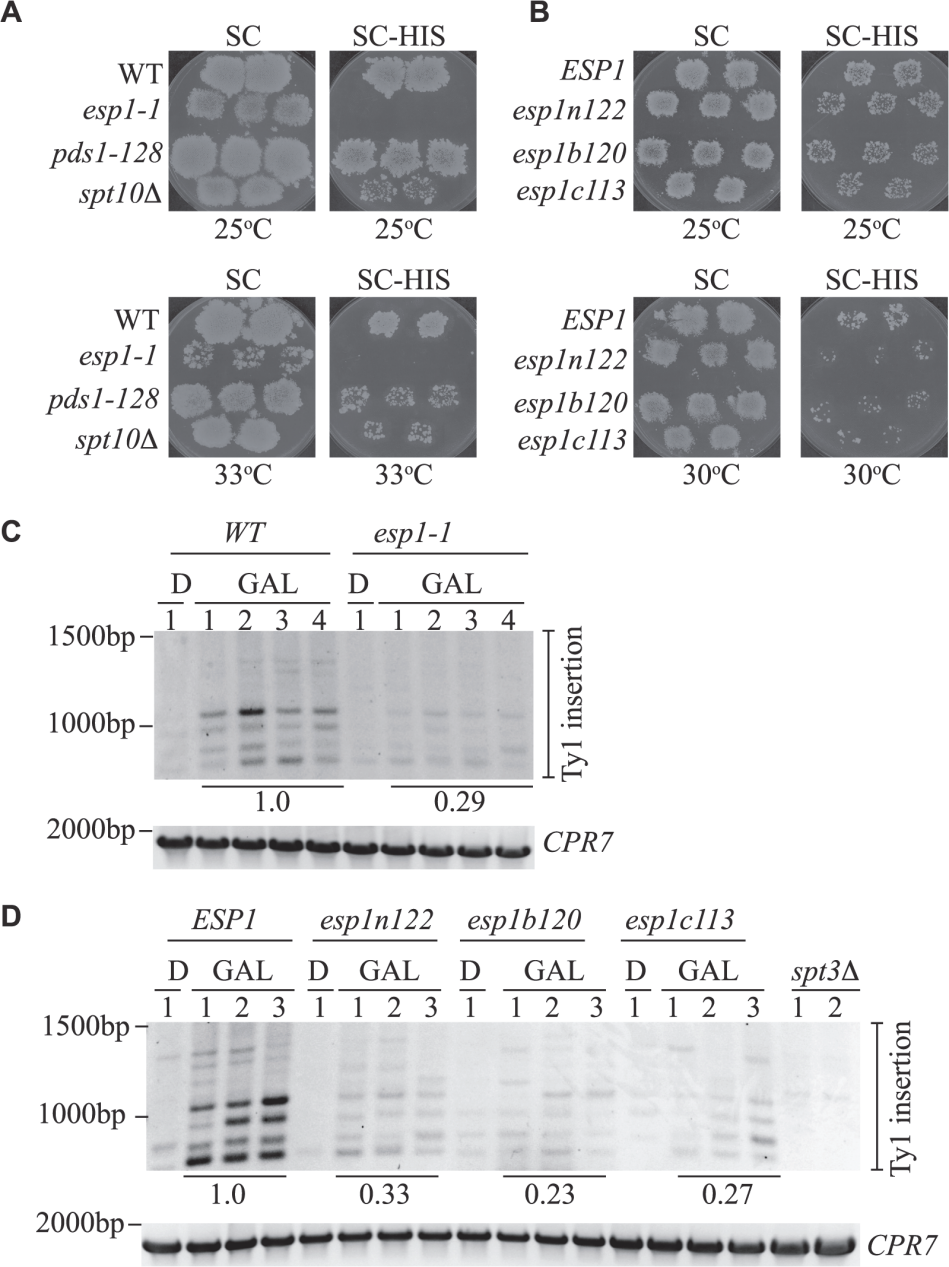
All Esp1 domains contribute to Ty1 transposition. (A,B) The indicated strains, carrying the *pGTy1-H3-mhis3AI–URA3* plasmid, were patched and induced to undergo transposition on galactose media at the indicated temperatures. After transposition, plates were replica plated to 5-FOA to remove the *URA3* plasmid as described in the Materials and Methods. Patches are shown after the final step of replica plating. The SC (minimal complete) plates are a control for growth and the colonies present on the SC-HIS plates represent insertion of the *Ty1-HIS3* element into the genome. (C,D) Triplicate isolates of indicated *esp1* ts alleles, carrying a *pGAL-TyH3mHIS3AI-URA3* plasmid (pJBe376) were induced to undergo transposition at semi-restrictive temperature (30°C) by growth in 2% galactose for 24 hours (GAL). One isolate was grown for 24 hours in 2% glucose (D) as a control. *SUF16* PCR analysis of the extracted yeast genomic DNA is shown and *CPR7* is a control PCR. All bands in the three lanes for each GAL grown isolate were quantified and summed, then compared to the wild type strain. The relative values of the mutants compared to wild type (value of 1.0) is shown. The amino acid mutations of the *esp1* alleles have been previously been published: *esp1n122* (N90S, C511F), *esp1b120* (K782E, I951T, I1040T), *esp1c113* (F1327L H1391Y) [26].

The Esp1 protein contains 1630 amino acids with the N-terminal 2/3 of the protein predicted to form tandem ARM/HEAT repeats and the protease catalytic domain at the C-terminus [9,63,64]. Thus far we had tested one allele of *esp1* with a single point mutation near the catalytic domain (*esp1-1*, P1404L). To further validate the function of Esp1 in Ty1 transposition, we acquired *esp1* ts alleles generated by creating mutations in three different regions of the protein—an N-terminal (*esp1n122*), middle (*esp1b120*) and C-terminal (*esp1c113*) allele [26]. The *esp1c113* allele does not have Scc1 cleavage activity at restrictive temperature [26]. Due to the ts nature of these alleles (restrictive temperature of 33°C), we developed two assays to test for Ty1 transposition at temperatures higher than 20°C, which is the temperature needed to induce endogenous Ty1 transposition. We used a modified version of a Ty1 transposition plate assay that employs an overexpression version of the *Ty1-H3mHIS3AI* element (*pGAL1*-*Ty1-H3mHIS3AI*), that can induce transposition at higher temperatures [40]. We included a step to remove the *pGAL-TyH3mHIS3AI-URA3* plasmid after transposition induction to avoid detecting HIS+ colonies due to Ty1 element recombination with the plasmid. In brief, patches of single isolate *ESP1* wild type and *esp1* mutant colonies carrying *pGAL1*-*Ty1-H3mHIS3AI* were induced on galactose media for 4 days to undergo transposition. After a series of replica plating steps, cell growth was monitored on minimal complete plates (SC) whereas Ty1 element insertion into the genome was measured by the presence of HIS+ colonies on SC-HIS plates (Fig. 9A, B). At 25°C, wild type cells produced a lawn of HIS+ colonies after *pGAL-TyH3mHIS3AI* induction whereas no HIS+ colonies were detected with *esp1-1* cells and reduced HIS+ colonies with the *spt10Δ* control strain (Fig. 9A). *The pds1-128* mutant, which has a higher restrictive temperature (37°C) than the *esp1* alleles, required growth at 33°C to display a transposition defect under these conditions (Fig. 9A). The additional *esp1* alleles (*n122*, *b120* and *c113*) all displayed a transposition defect at 30°C, but not 25°C (Fig. 9B).

We also analyzed Ty1 insertion events upstream of the *SUF16* locus by inducing the *pGAL-TyH3mHIS3AI* plasmid for 24 hours while growing the *esp1* ts alleles at semi-permissive temperature (30°C). We found that after expression of the *pGAL-TyH3mHIS3AI* plasmid for 24 hours at 30°C, we were able to detect insertion of Ty elements at the *SUF16* locus in a wild type strain (Fig. 9C, D). We first tested the *esp1-1* allele that has a 2-fold reduction in transposition at 20°C and found that the Ty insertion events were reduced ∼3-fold (0.29) compared to wild type (Fig. 9C). Next we tested the three *esp1* alleles that represent three regions of the Esp1 protein (*esp1n122*, *esp1b120* and *esp1c113*) and found that *esp1b120* was reduced ∼4-fold whereas *esp1n122* and *esp1c113* were reduced ∼3 fold compared to wild type (Fig. 9D). Therefore, no specific domain of Esp1 contributes solely to Ty1 transposition activity and a cohesin cleavage defective version of Esp1 (*esp1c113*) also has transposition defects.

### The mitotic exit network impacts Ty1 transposition

**Fig 10:**
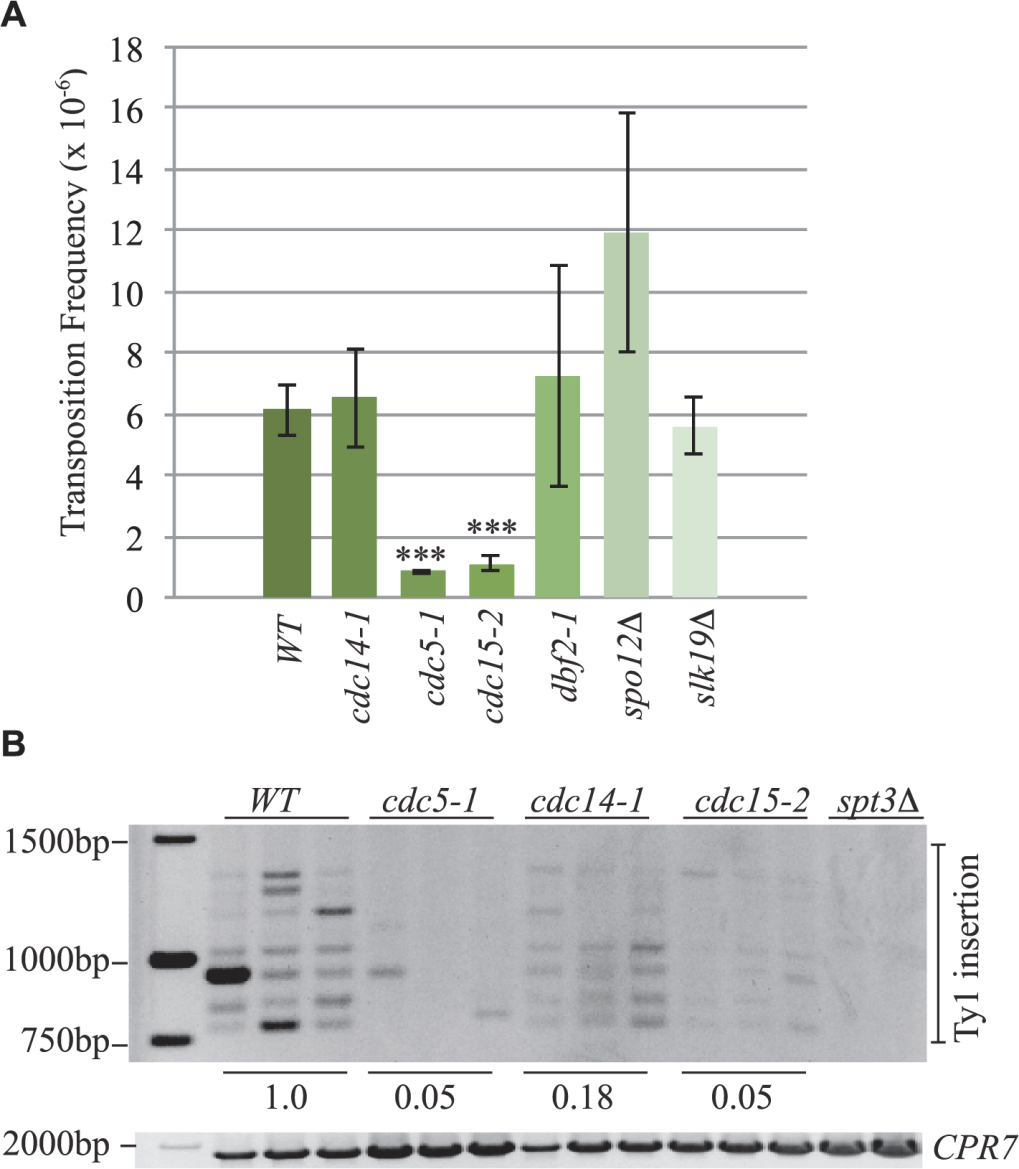
The mitotic exit network has a role in Ty1 transposition. (A) Transposition frequency of wild type (*WT*), *cdc14-1*, *cdc5-1*, *cdc15-2*, *dbf2-1*, *spo12Δ* and *slk19Δ* cells carrying a plasmid with a marked Ty1element (pBDG922). Mutants with transposition frequencies that are significantly different from WT are shown with three asterisks (p<0.0005). (B) PCR analysis of yeast genomic DNA extracted from indicated strains grown in triplicate for 3 days at 20°C to induce transposition. Upper panel is a *SUF16* PCR assay whereas the lower panel is a control PCR for the *CPR7* locus to demonstrate that yeast genomic DNA was present in each sample. Quantification of Ty1 insertion events is shown relative to WT as described in the Materials and Methods.

Cleavage of cohesin by separase is important for triggering the end of the cell cycle [65]. Esp1 is also part of the “FEAR” pathway that promotes early anaphase release of the Cdc14 phosphatase from its inhibitor Cfi1/Net1 in the nucleolus [18]. This function for separase does not require its proteolytic activity [19]. The mitotic exit network (MEN) fully releases Cdc14 from the nucleolus after anaphase to down-regulate Cdk activity and promote mitotic exit [66]. The MEN is a Ras-like GTPase cascade that includes the Cdc5, Cdc15 and Dbf2 protein kinases [66]. We tested if components of the FEAR and MEN pathway have a role in Ty1 transposition by performing a quantitative transposition assay at 20°C in FEAR (*spo12Δ*, *slk19Δ*, *cdc5-1*) and MEN (*cdc5-1*, *cdc15-2*, *dbf2-1*) mutants as well as the *cdc14-1* mutant. We found that the Ty1 transposition frequency was reduced by 7-fold in *cdc5-1* mutants and 5.5-fold in *cdc15-2* mutants whereas none of the other mutants had a statistically significant change in transposition frequency compared to wild type (Fig. 10A). Consistent with our quantitative transposition data, Ty1 insertion events upstream of the *SUF16* locus were also dramatically reduced in *cdc5-1* and *cdc15-2* mutants (Fig. 10B). Although we could detect a clear banding pattern of *SUF16* Ty1 insertion events in the *cdc14-1* mutant, the intensity was significantly lower than wild type. Therefore Cdc14 may impact specific Ty1 insertion sites but not overall Ty1 transposition. Our data suggests that the Cdc5 and Cdc15 kinases are required for optimal Ty1 transposition.

## Discussion

We present here a combined study of both genetic and physical interactions that uncovered a novel role for Esp1 in Ty1 retrotransposition. Using SGA technology, we identified genes that upon loss of function (SL) or increased dosage (SDL) adversely impact *esp1-1* viability. As expected, genes with a cell cycle function were uncovered in our *esp1-1* SL and SDL screens. However, genetic interactions were enriched for alleles with roles in other biological pathways, including the regulation of Ty1 transposition. Further, using mass spectrometry analysis, we made the discovery that Esp1 interacts with Ty1-IN encoded by the Ty1 retrotransposon. Despite extensive studies of Ty1 transposition, no host factor has been identified that interacts with Ty1-IN. We confirmed that Esp1 interacts with Ty1-IN and that both Esp1 and Pds1 are required for wild type levels of Ty1 element mobility and insertion frequency. The identification of proteins that regulate the chromosome division machinery with a role in Ty1 transposition concurs with a previous analysis that identified enriched GO terms from independent screens for genes that either inhibit or promote Ty1 transposition [42]. In this analysis, the enriched GO terms included as “M phase” and “G2/M transition of the mitotic cell cycle” which fits well with our data that Esp1 mediates Ty1 transposition.

### Esp1 interacts with Ty1-IN

Only two proteins—the TFIIIB subunit Bdp1 and the Isw2 chromatin remodeling protein have been shown to disrupt the prototypical 80bp periodicity of the Ty1 target site when mutated [67,68]. However, neither of these factors was demonstrated to interact with Ty1-IN. Our mass spectrometry data identified an enrichment of both Ty1-IN and Ty1-RT peptides in the Esp1 versus mock purification (Fig. 3A). The interaction between Esp1 and Ty1-IN was confirmed by two independent co-IP assays (Fig. 3B, C). However, we were not able to confirm the putative interaction of Esp1 with Ty1-RT. As well, Ty1 cDNA levels are not affected in *esp1-1* mutants suggesting that Esp1 is not required for the reverse transcription of Ty1 mRNA (Fig. 7A).

The Esp1-Ty1-IN interaction occurs in Log, G1 and G2/M cells but is minimal in S phase cells (Fig. 4). The efficient concentration of Esp1 in the nucleus in late G2 phase depends on Pds1 [38]. We find that both *esp1-1* and *pds1-128* mutants have decreased Ty1 mobility and frequency of Ty1 insertions upstream of the *SUF16* locus (Fig. 6A, 8B, C). However, Pds1 does not appreciably interact with Ty1-IN and is not required for Esp1 to interact with Ty1-IN (Fig. 3B, 5B). Therefore, we suspect that the Ty1 insertion defect in the *pds1-128* mutant is due to a reduced efficiency of Esp1 localization to the nucleus in this mutant [38]. Although both Esp1 and Ty1-IN are nuclear localized proteins, it still remains to be tested if the interaction between Esp1 and Ty1-IN occurs specifically in the nucleus [38,69–71]. Ty1 mobility is modestly reduced in *esp1-1* and *pds1-128* mutants (2 and 6-fold, respectively), compared to the 5 to 50-fold reductions detected in Ty1-integrase nuclear localization mutants [69,71]. The relatively modest decrease Ty1 mobility that in *esp1* and *pds1* mutants may be due to the use of ts alleles that do not completely eliminate function, or because another host factor is also required for Ty1 element insertion.

### Is removal of cohesin required for Ty1 transposition?

Our data suggests that removal of cohesin by separase enables insertion of Ty1 elements upstream of tRNA genes. The majority of cohesin mutants that we tested displayed higher levels of transposition frequency and Ty1 element insertion and the transposition defect of the *esp1-1* mutant is at least partially rescued when combined with the *scc1-73* mutant (Fig. 6A, 8). We also find that an allele of *esp1* (*c113*) that cannot cleave Scc1 at restrictive temperature has reduced transposition efficiency (Fig. 9B, D). Finally, a version of *ESP1* that lacks the entire protease domain cannot rescue the *esp1-1* transposition defect (Fig. 6B). However, two pieces of data suggest that the cleavage of cohesin by separase cannot be the only function for Esp1 in mediating Ty1 transposition. The first is that Ty1-IN physically interacts with Esp1 and this interaction is enhanced in metaphase cells (Fig. 3, 4). The second is that expression of a catalytically inactive version of *ESP1* is partially capable of restoring Ty1 transposition mobility to an *esp1-1* mutant (Fig. 6B). One way to explain the data is that two activities of Esp1 contribute to efficient transposition—one is removal of cohesin and the other is a chromatin targeting function based on the physical interaction of Ty1-IN with Esp1[8,9,26].

### Scc3 may not impact Ty1 element insertion

We noticed a striking increase in Ty1 cDNA in the *scc3-1* mutant (4 to 5-fold) compared to no increase in the other cohesin mutants upon galactose induction of a Ty1 element (Fig. 7A). Although we also detected a corresponding increase in Ty1 transposition events, based on the *Ty1-H3mHIS3AI* transposition assay (Fig. 6A), we did not see an increase in Ty1 element insertion events upstream of *SUF16*. Therefore, the increase in HIS3+ colonies could be due to recombination from higher levels of Ty1 cDNA in the *scc3-1* mutant rather than an increase in transposition. Scc3 is not part of the core cohesin molecule but associates with Scc1 in a regulatory manner. The *scc3-1* mutant prematurely separates sister chromatids, which is a similar phenotype to the *scc1-73* and *smc1-259* mutants [72,73]. However Scc3 is primarily thought to be involved in cohesin establishment in S phase and specific mutations in Scc3 reveal that it also has an anti cohesin-establishment activity as well [11,74]. The removal of Scc3 does not affect cohesin ring stability, which may explain why there is no increase in Ty1 insertion events upstream of *SUF16* in the *scc3-1* mutant (Fig. 8C, [74]).

### Ty1 element insertion upstream of tRNA genes is not due to cohesin loading

Cohesin loads onto chromosomes at sites defined by the Scc2/4 cohesin loading complex, correlating with tRNA sites and other genes transcribed by RNA pol III [75,76]. After cohesin loads, it moves to sites of convergent transcription [3,4]. However, the creation of a synthetic chromosome III lacking all tRNA genes suggests that tRNA genes are not absolutely required for cohesin loading [77]. The cohesin binding pattern was similar on the synthetic chromosome compared to native chromosome III suggesting that in the absence of tRNA genes, secondary loading sites may become prevalent [77]. Ty1 elements preferentially integrate upstream of genes transcribed by RNA polymerase III, such as tRNA genes, in a loosely defined window ranging from about 80bp to ∼750bp [60,78–80]. Our data suggests that the loading of cohesin at tRNA genes is not required for Ty1 insertion because we detect increased Ty1 mobility and insertion events in cohesin mutants (Fig. 6, 8). Although we did observe a weak interaction between Scc1 and Ty1-IN, Scc1 was not required for the Esp1-Ty1-IN association (Fig. 3B, 5C). Therefore the Scc1-Ty1-IN interaction is likely indirect and mediated by Esp1 that is bound to the cohesin complex. We analyzed high resolution mapping of cohesin binding sites in metaphase arrested cells and found that, as expected cohesin, does not bind the *SUF16* tRNA site or most other tRNA sites unless they are located very near the centromere (a high density cohesin binding region) [4]. Although *SUF16* is not bound by cohesin, the gene upstream of this tRNA site (YCR016W) is [4]. One intriguing possibility is that Esp1 clears cohesin from the region upstream of tRNA genes to enable Ty1 insertion and the interaction with Ty1-IN serves to target it to this location. Localized removal of cohesin by separase has been demonstrated in both *S*. *cerevisiae* and *S*. *pombe* in response to DNA damage [37,81].

### Esp1 is well positioned to mediate Ty1 insertion during mitosis

A recently published paper noted that tRNA genes are enriched in the pericentromere—a region of 30 to 50kb surrounding the centromere that is also enriched in cohesin and condensin [82–84]. tRNA genes, condensin and a protein called Cbf5/Dyskerin were found to tether pericentric chromatin to the spindle axis in mitosis [84]. This data fits nicely with the fact that Esp1 localizes to and promotes stability of the elongating spindle in mitosis and our data demonstrating an enrichment of the Esp1 Ty1-IN interaction in mitosis (Fig. 4, [15,38]). Cbf5/Dyskerin is a component of the H/ACA snoRNP pseudouridylase complex that catalyzes the conversion of uridine to pseudouridine in nascent tRNA [85]. We identified another component of the H/ACA snoRNP pseudouridylase complex, Gar1, in our *esp1-1* SDL screen (S2 Table). Notably, the *SUF16* locus, which is a hotspot for Ty1 transposition, is located within the pericentromere of chromosome III. Therefore Esp1 may have a specific role in targeting Ty1-IN to tRNA genes within the pericentromere.

Our data also points to a possible connection between Ty1 transposition and events that occur after chromosome segregation. We find that two kinases, Cdc5 and Cdc15, that function to promote mitotic exit via the FEAR and MEN pathways promote Ty1 transposition (Fig. 10). The role of the Cdc5 kinase in mitotic exit is complex because Cdc5 activates the MEN pathway by phosphorylation of many of its components [66]. Importantly, Cdc5 also phosphorylates Scc1 and this phosphorylation is important for Scc1 cleavage by separase, therefore the lack of transposition in the *cdc5-1* mutant could be due to inefficient cohesion dissolution [9,86]. We were unable to conclusively determine if the Cdc14 phosphatase is important for transposition because our quantitative assay did not show a significant difference in HIS+ colonies but our *SUF16* assay showed a reduced efficiency of Ty1 insertion (Fig. 10). A potential link between Ty1 transposition and the FEAR pathway is intriguing because the FEAR pathway controls segregation of the nucleolus, which is where tRNA genes are clustered [16,18,19,66,87,88].

The conservation of retroviral and retrotransposon integrases suggests that the cellular mechanisms used to insert Ty1 cDNA into the *S*. *cerevisiae* genome may be conserved with retroviral cDNA insertion into eukaryotic genomes. Interestingly, the dominant binding partner of HIV-1 IN in human cells is lens epithelium derived growth factor (LEDGF) and the LEDGF IN binding domain has high structural similarity to HEAT/ARM repeats [89]. The N-terminal two thirds of separase is predicted to form tandem HEAT/ARM repeats suggesting this might be a common motif for IN binding [63,64]. Our study, which reports the first physical interaction of a yeast host protein with Ty1-IN, is an important step towards understanding the mechanism of targeting retroviral cDNA into the host genome.

## Materials and Methods

### Yeast strain construction

*S*. *cerevisiae* strains used in this study are listed in S4 Table. All strains were grown at 25˚C unless otherwise indicated. To create the *esp1-1*::*natR* mutation in the SGA starting strain, a C-terminal fragment was amplified from *esp1-1* (W303 strain background, gift from Yanchang Wang) [25]. This fragment included the P1404L (bp4211 C→T) mutation, approximately 200 bp downstream of the stop codon and an additional 25 bp overlapping the *TEF* promoter in p4339 [27]. Concurrently, the *natR* cassette from p4339 was also amplified to include 45 bp immediately downstream of the *ESP1* ORF. Both amplicons were transformed simultaneously into Y7092. Successful double recombination was confirmed in three ways: (1) growth on yeast peptone dextrose (YPD) media containing clonNAT (2) temperature sensitivity at 37°C (3) sequencing the *esp1-1*::*natR* mutation (Macrogen).

To create YM2377, a single copy of *ESP1/YGR098C* was disrupted by homologous recombination with the *hphMX4* marker in BY4743 as described [90]. To generate YM2390 a *LEU2-CEN* plasmid carrying the *esp1n122* ts alleles was transformed into YM2377, sporulated and haploid isolates containing *esp1ΔhphMX4* and the *pesp1n122-LEU2-CEN* plasmid were isolated. YM2392 and YM2400 were generated in a similar manner after transformation of YM2377 with *LEU2-CEN* vectors carrying the *esp1b120* and *esp1c113* ts alleles respectively whereas YM2396 was created by transformation of YM2377 with a wild type *ESP1-LEU2-CEN* plasmid. The *esp1* alleles have been previously published [26].

### Plasmid construction

The *pESP1-LEU2-CEN* vector contains full length *ESP1* and its promoter and was a kind gift from Dr. Steven Reed [26]. The pESP1C1531A vector was constructed by cloning an NdeI fragment from a GAL-FLAG-ESP1 (C1531A)-CBD construct [kindly provided by Dr. Frank Uhlmann [9]] that contains the C1531A mutation, into *pESP1-LEU2-CEN* that had been digested with NdeI. The pESP1-1112 mutation was created using the same cloning strategy but the NdeI fragment inserted in backwards, resulting in termination of wild type Esp1 sequence at amino acid 1112, the introduction of 24 new amino acids until a stop codon was generated.

### SGA screens

SGA screens have been previously described in detail [20,27,91]. Using a Singer RoToR HDA robot, MATα *esp1-1*::*natR* was mated to both the haploid deletion collection of nonessential alleles (BY4741, MATa, KanMX4) and the 2 micron *pGAL1/10-GST-ORFX* overexpression array (Open Biosystems) [20,29]. For SL and SDL screens respectively, either double mutants (*esp1-1*::*natR geneXΔKanMX4*) or *esp1-1* carrying *pGAL1/10-GST-ORFX* were obtained through sporulation of heterozygous diploids with at least three colonies of each genotype being compared. *MAT* a cells of interest (*esp1-1*::*natR* in combination with *geneXΔKanMX4* or *pGAL1/10-GST-ORFX* for the SL or SDL screens, respectively) were isolated through a series of replica pinning steps. Growth on the final selective media (clonNAT + G418 dextrose plates for the SL screen; SC-URA + clonNAT) was examined at 25°C, with plates imaged using a flatbed scanner. Balony software was used to measure spot sizes, determine cut-off values for genetic interactions and define strains the showed statistically significant changes in growth rate [30]. For SL interactions, cut-off values were defined for each screen by determining three standard deviations from the mean of the ratios of the double mutant to single mutant growth rates. Double mutant strains that met the cut-off and showed significant changes in growth relative to the corresponding single mutant control (one-tailed students’ t-test; p < 0.05; n = 3) were considered as genetic interactions. *esp1-1* SDL interactions were similarly analyzed by comparing growth of *esp1-1*::*natR pGAL1/10-GST-ORFX* on dextrose versus galactose.

### Confirmation of SDL interactions

Plasmids carrying genes identified as *esp1-1* top SDL interactions (causing a growth rate < 0.3 of control) were rescued from yeast and retransformed into both wild type and *esp1-1*::*NAT* strains. Transformants were then grown overnight to log phase (OD_600_ of 0.5–1.0) in SC media depleted for uracil + 2% raffinose + 0.1% dextrose media. Strains were then diluted to an OD_600_ of 0.1, and subsequently serially diluted four times by a factor of 1:5, all in SC-URA + 2% raffinose. Dilutions were spotted onto either SC-URA + 2% dextrose or SC-URA + 2% galactose plates, and incubated for four days (S1 Fig). Genes that caused a growth defect in *esp1-1* but not wild type cells when overexpressed on galactose were scored as SDL or synthetic dosage sensitive.

### Affinity IP and mass spectrometry

Esp1-13Myc was purified using a one-step Myc-tag approach based on Field *et al* [92]. Briefly, 1L each of WT and *ESP1-13Myc*::*KanMX6* strains were grown to an OD_600_ of ∼0.9. Cells were lysed in 100mM Tris-HCl pH 7.5, 150mM NaCl, 0.1% Tween 20, 1% NP-40, 10% glycerol, 5mM EDTA, 1mM DTT, 1mM phenylmethylsulfonyl fluoride, 10mM chloroacetamide and a 1x protease inhibitor cocktail by glass bead beating. Lysates were cleared by centrifugation at 10,000 rpm, 4°C for 15 minutes. IPs were performed using a 9E10 anti-Myc antibody cross-linked to Sepharose (MMS-150P, Covance). 400μl beads (bed volume) were mixed with each lysate for four hours at 4°C and washed five times in 100mM Tris-HCl pH 7.5, 500mM NaCl, 0.05% Tween 20, 0.5% NP-40, 10% glycerol, 5mM EDTA and 1mM DTT. Proteins were eluted twice with 200μL of SDS loading sample buffer without reducing agent prior to TCA precipitation. Proteins were then separated by SDS-PAGE followed by in-gel digestion for mass spectrometry analysis. The protein gel was first incubated in fixation buffer (5% acetic acid, 47.5% methanol) with coomassie Blue G for 30 minutes following by a four-hour wash in H_2_O. Gel pieces from each sample (Esp1-IP and control) were divided into five fractions: three fractions above 55 kDa; one fraction bellow 55 kDa without immunoglobulins; and one fraction containing the immunoglobulin G heavy chain and light chains (55kDa and 25kDa bands). In-gel trypsin digestion was based on Shevchenko *et al*. with minor changes [93]. Digestion buffer contained 50mM ammonium bicarbonate, and gel pieces were dehydrated using ethanol. Gel pieces were incubated at 56°C for 45 minutes in 10mM DTT followed by a 30-minute incubation in 55mM chloroacetamide at room temperature. Peptides were extracted once with 0.5% acetic acid, twice with 30% acetonitrile, 0.5% acetic acid and twice with 100% acetonitrile. Dried peptide-extraction samples were resuspended in 0.5% acetic acid solution and acetified to a pH below 2 using 100% acetic acid. Peptide samples were then purified by solid phase extraction on C-18 stage tips [94–96].

Purified peptides were analyzed using a linear-trapping quadrupole—Orbitrap mass spectrometer (LTQ-Orbitrap Velos; ThermoFisher Scientific) coupled to Agilent 1200 Series high-performance liquid chromatography using a nanospray ionization source that included a 100-μm-inner diameter fused silica trap column packed with 5 μm-diameter Aqua C-18 beads (Phenomenex, www.phenomenex.com), 50-μm-inner diameter fused silica fritted analytical column packed with 3 μm-diameter Reprosil-Pur C-18-AQ beads (Dr. Maisch, www.Dr-Maisch.com) and a 20-μm-inner diameter fused silica gold coated spray tip. Fractions from each sample were run at a 60-minute high-performance liquid chromatography gradient from 0.5% acetic acid to 0.5% acetic acid and 26% acetonitrile). The LTQ-Orbitrap was set to acquire a full-range scan at 60,000 resolution from 350 to 1600 Th in the Orbitrap and to simultaneously fragment the top ten peptide ions in each cycle in the LTQ (minimum intensity 1000 counts). Singly charged ions were excluded and parent ions were then excluded from MS/MS for the next 30 sec. The Orbitrap was continuously recalibrated using lock-mass function [97].

### Analysis of mass spectrometry data

Centroided fragment peak lists were processed with Proteome Discoverer v. 1.2 (ThermoFisher Scientific) followed by a Mascot analysis (2.3.0, Matrix Science) against a *Saccharomyces cerevisiae* protein database (www.yeastgenome.org; 05012010, 6147 protein sequences) with the following parameters: peptide mass accuracy 10 parts per million; fragment mass accuracy 0.6 Da; trypsin enzyme specificity; fixed modifications—carbamidomethyl; variable modifications—methionine oxidation, lysine acetylation, serine/threonine/tyrosine phosphorylation; ESI-TRAP fragment characteristics. Only those peptides with IonScores exceeding the individually calculated 99% confidence limit (false positive discovery rate < = 1%) were considered as accurately identified. Candidate Esp1-interacting proteins were arbitrarily selected if at least three peptides were identified and if peptides were at least four times more abundant in the IP versus the mock-IP samples (S3 Table).

### Co-immunoprecipitation

Fig. 3A—GFP-tagged Esp1, Pds1 and Scc1 and untagged wild type cells (BY4741 strain background) were transformed with *pGAL1-Ty1-H3* (pJEF724, generous gift from Dr. Jef Boeke) and grown overnight to log phase in SC-URA + 2% raffinose + 0.1% dextrose. 100mL of cells were then diluted to an OD_600_ of 0.02, resuspended in SC-URA + 2% galactose to induce *pGAL1-Ty1-H3* expression for 24 hours. Cells were harvested, washed with ice cold H_2_0 and resuspended in lysis buffer (50mM Tris-HCl pH 7.5, 0.1% NP40, 150mM NaCl, 0.5mM EDTA and protease inhibitors) on ice followed by addition of glass beads and lysed. Lysates were centrifuged at 12 000 xg for 5 mins, and supernatant retained as whole cell lysate. Protein concentration was quantified by Bradford assay. 5mg of lysate were first incubated with 5μl of Bab-20 beads (ChromoTek) at 4°C for 1 hour to reduce non-specific binding. The supernatant was removed and incubated with GFP-Trap_A beads (ChromoTek) at 4°C for 1.5 hours. Beads were then washed four times with wash buffer (50mM Tris-HCl pH 7.5, 0.5% Triton, 400mM NaCl, 0.5mM EDTA) and finally resuspended in 20μl 2X sodium dodecyl sulfate (SDS) loading buffer containing 200mM β mercaptoethanol. 10μg of Ty1-IN and 80μg of GFP tagged proteins were loaded on the immunoblot for whole cell lysates whereas 5μl of the IP was loaded. Samples were run on 8% SDS polyacrylamide gels and transferred to polyvinylidene difluoride. Membranes were probed with either anti-GFP (1:500 Roche) or anti-IN [1:1000, 8B11, J. Boeke [98]]. Blots were imaged with a ChemiDoc MP (Biorad).

Fig. 3C—Esp1-13Myc cells harboring pGAL-GFP-LacZ-Ty1-IN [kind gift of Anita Corbett, [70,71]] and pGAL-GFP-lacZ vector control were grown to log phase in SC-URA + 2% raffinose + 0.1% dextrose and diluted down to an OD_600_ of 0.08 in SC-URA + 2% galactose to induce Ty1 expression for 24 hours. Esp1-13Myc and untagged (No Tag) cells carrying pGAL-GFP-lacZ grown in galactose and pGAL-GFP-LacZ-Ty1-IN grown in 2% dextrose were used as controls. Cell lysates and GFP purification were performed as described above. Membranes were probed with anti-GFP (Roche) and anti-c-Myc (9E10, 1:2000, Roche).

Fig. 4 - The Esp1-GFP cell cycle IP (Fig. 4) was performed in a similar manner with the following changes. Esp1-GFP cells carrying *pGAL1-Ty1-H3* were grown in galactose for 20 hours, fresh 2% galactose added and grown for another 4 hours, then arrested with either α-factor (5μg/mL) for 2 hours, HU (200mM) for 3.5 hours, or Nz (15μg/ml) for 3 hours prior to cell lysis.

Fig. 5B—*TetO*_*7*_ *-PDS1* Esp1-GFP and *TetO*_*7*_ *-PDS1* cells were transformed with *pGAL1-Ty1-H3-LEU2* (pX87, kind gift from Dr. Jef Boeke). Cells were grown overnight in SC-LEU + 2% raffinose + 0.1% dextrose, then diluted to an OD_600_ of 0.02 and induced in SC-LEU + 2% galactose for 20 hours. Next, fresh 2% galactose was added and cells grown for an additional 4 hours before treatment with 100μg/ml Doxycycline (Sigma) for 1hour. Cell lysates and GFP purifications were performed as described above.

Fig. 5C—*TetO*_*7*_ *-SMC1* Esp1-GFP and *TetO*_*7*_ *-SMC1* cells were treated as described above except that 100μg/mL Doxycycline was added for six hours prior to cell lysis.

### Tet-PDS1 quantitative PCR

The *TetO*_*7*_ *-PDS1* Esp1-GFP strain were grown to log phase in SC-URA with 2% glucose, 100μg/ml Doxycycline (Sigma) was added and time points taken at 15, 30, 60,120 and 240 min. Approximately 1x10^7^ cells were spun down and stored at -80°C. Total RNA was prepared with Qiagen RNeasy kit and 1μg RNA was used for cDNA synthesis with Superscript VILLO cDNA synthesis kit (Invitrogen). Real-time PCR was performed in duplicate with 2μl diluted cDNA (1:50) and Power SYBR Green Master Mix (Invitrogen). A standard two-hour comparative PCR analysis was performed using a 7500 Real Time PCR system (Applied Biosystems). Primers were designed to amplify *PDS1* (OVM866 5’- GGCAGCAAAAGACAACAATAG-3’ and OVM876 5’-CCCTACTACGACTGCGAGAAA-3’) and *TAF10* as an internal control [(OVM695 5’- GGCGTGCAGCAGATTTCAC-3’ and OVM696 5’- TGAGCCCGTATTCAGCAACA-3’ [99]]. Relative transcript values and error bars were quantified as described [Guide to Performing Relative Quantitation of Gene Expression Using Real-Time Quantitative PCR, Applied Biosystems (www.appliedbiosystems.com)] using 7500 Software V.2.0.6.

### Ty1 quantitative transposition assay

This assay was based on the Ty1-his3-AI mobility assay performed in [42]. Strains were transformed with a *URA3 CEN* based plasmid that expresses a marked Ty1-his3-AI element at endogenous levels (pBDG922, a kind gift from Dr. David Garfinkel). Single colonies were isolated at 25°C and patched. 2000 cells were inoculated into 1mL of SC-URA3 media in triplicate and grown for four or five days at 20°C until saturation. 1x 10^7^ cells were plated on SC-URA-HIS plates to test for transposition frequency and 200 cells were plated on SC-URA plates to test for viability. Cells were grown on plates at 25°C for 2 days. The frequency of Ty1-his3-AI mobility was calculated according to this formula: # colonies on the SC-URA-HIS plate/1 x 10^7^ cells divided by #colonies on the SC-URA plate/200.

### Ty1 qualitative plate transposition assay

Yeast strains were transformed with a *pGAL-TyH3mHIS3AI-URA3* plasmid (pJBe376, kind gift from Jef Boeke) and were plated on synthetic complement (SC) media minus uracil (SC-U) and grown at 25°C. Single colony transformants were patched onto SC-U 2% glucose media and grown for 2 days at 25°C. Next, plates were replica plated at onto SC-U containing 2% galactose and incubated for 1 day at 25°C, then either kept at 25°C for 3 additional days or incubated at 30°C or 33°C for 3 additional days. From this point onward, plates were kept at the same temperature as for the final 3 days of galactose induction. After galactose induction, plates were sequentially replica plated onto 2% glucose plates containing: (i) SC-U media, (ii) yeast peptone dextrose (YPD), (iii) SC-U plus 1.2 g/L of 5-fluoroorotic acid (5-FOA), and (iv) SC lacking histidine (SC-H) and SC complete. For each of the final four stages of replica plating, patches were grown until confluent.

### Ty1 cDNA analysis

Ty1 cDNA analysis for Fig. 7A was performed as described in [40] with the following modifications. Strains were transformed with the pGTy1-H3-mhis3AI plasmid [[56], kind gift from Dr. Jef Boeke] and induced with galactose for 24 hours. Yeast genomic DNA was extracted and 4μg of DNA digested with *Afl II* to release a ∼2.4kb fragment of Ty1 cDNA containing the *HIS3* gene. Digestions were run on a 1% agarose gel, transferred to a nylon membrane and probed with an 856bp fragment of the *HIS3* gene including the promoter region. The *HIS3* probe was radiolabelled with ^32^P-α-dATP using a DecaLabel DNA Labeling Kit (ThermoScientific). Ty1 endogenous cDNA analysis (Fig. 7B) was performed as described [47,100] with the following modifications. Cells were grown for 2 days at 20°C. Yeast genomic DNA was extracted and 10μg of DNA digested with *PvuII* to release a ∼2kb fragment of Ty1 cDNA. Blots were probed with a radiolabeleld 1517bp PvuII/SnaBI C-terminal fragment of the Ty1 element.

### SUF16 PCR assay

The *SNR33* and Ty1 PCR primers as well as the *CPR7* primers have been previously described [42]. 100ng of DNA was used for each *SUF16* PCR assay and 5ng of DNA was used for the *CPR7* PCR assay.

### pGAL-Ty1 SUF16 assay

*esp1* ts alleles were transformed with a *pGAL-TyH3mHIS3AI-URA3* plasmid (pJBe376). Three independent transformants were grown overnight in SC-URA-LEU + 2% raffinose + 0.1% dextrose. One isolate (#1) was diluted to an OD_600_ of 0.05 in SC-URA-LEU + 2% glucose (D) and three isolates (#1–3) were induced to undergo transposition by diluting to an OD_600_ of 0.05 in SC-URA-LEU + 2% galactose (GAL) for 24 hours. All cells were grown at semi-restrictive temperature (30°C). Cells were pelleted, yeast genomic DNA extracted and a *SUF16* and control *CPR7* PCR assay performed.

## Supporting Information

S1 Fig Confirmation of *esp1-1* SDL hits. Plasmids of interest were transformed into both WT and *esp1-1* strains. Logarithmically growing cells were serially diluted and plated onto dextrose (control) or galactose containing media to confirm growth defect when overexpressed in *esp1-1*.
(PDF)

S2 Fig Quantification of Gene Ontology networks. GO terms were clustered using the ClueGO [36] plugin for Cytoscape [35] using a Kappa value of 0.3. For each term, the number of genes found in the corresponding screen are shown relative to the total number of genes assigned to that term. (A) SL screen interactions, (B) SDL screen interactions.
(PDF)

S1 Table Genes identified in *esp1-1* SL screen. (DOCX)

S2 Table Genes identified in *esp1-1* SDL screen. (DOCX)

S3 Table Affinity mass spectrometry peptides identified for Esp1 purification. (DOCX)

S4 Table Yeast strains used in this study. (DOCX)
