# Supplementary figures and images for: A Role for the Budding Yeast Separase, Esp1, in Ty1 Element Retrotransposition
Source: PLoS Genet. 2015 Mar 30;11(3):e1005109. doi: 10.1371/journal.pgen.1005109 (PMC4378997; doi:10.1371/journal.pgen.1005109)

Figure S1

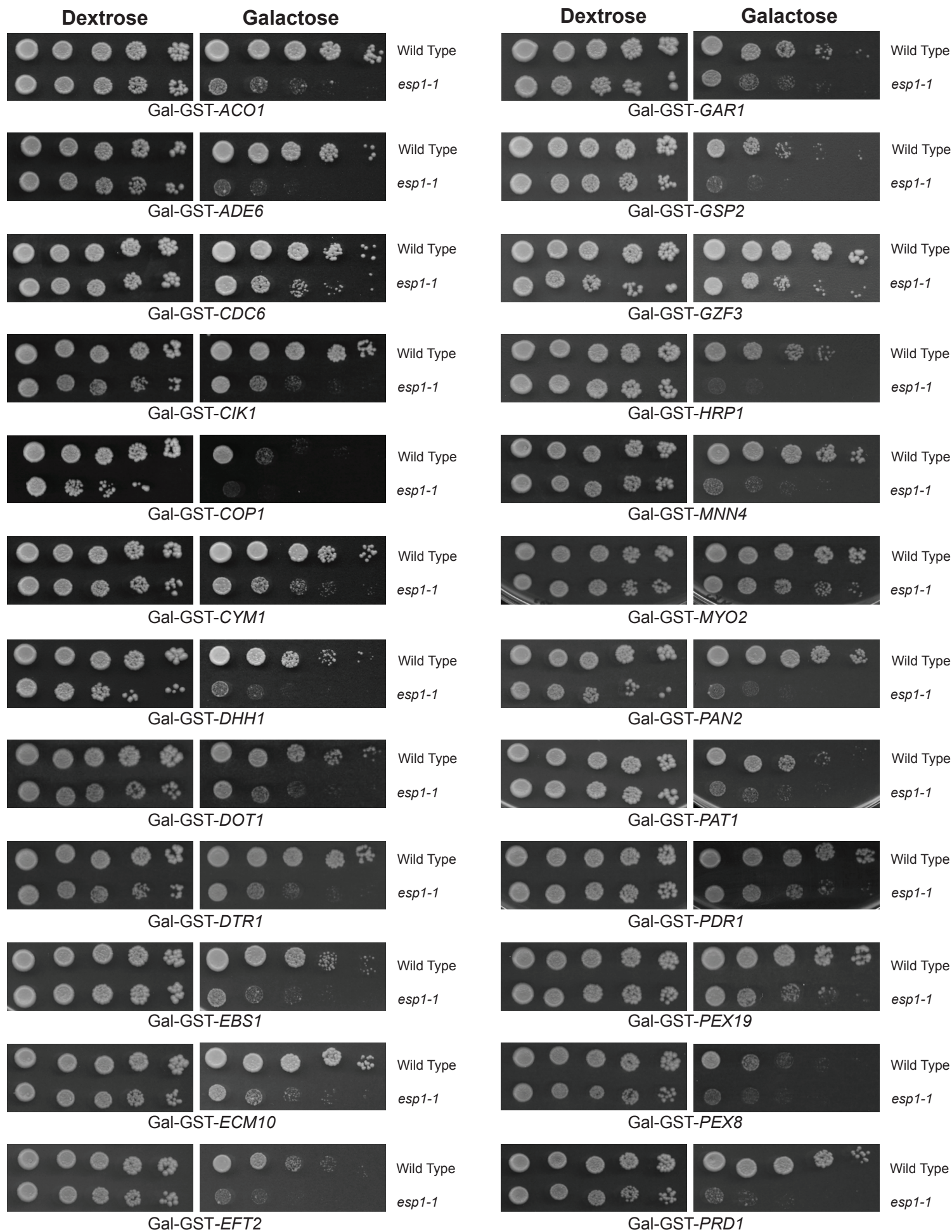

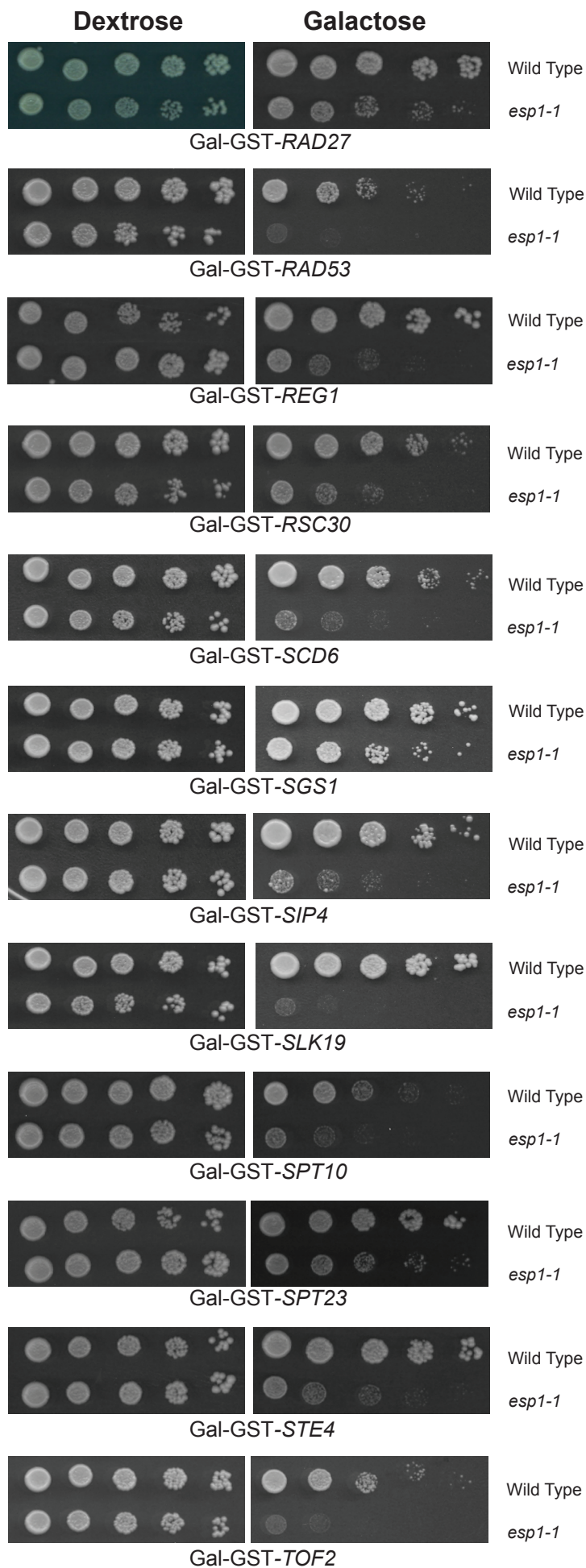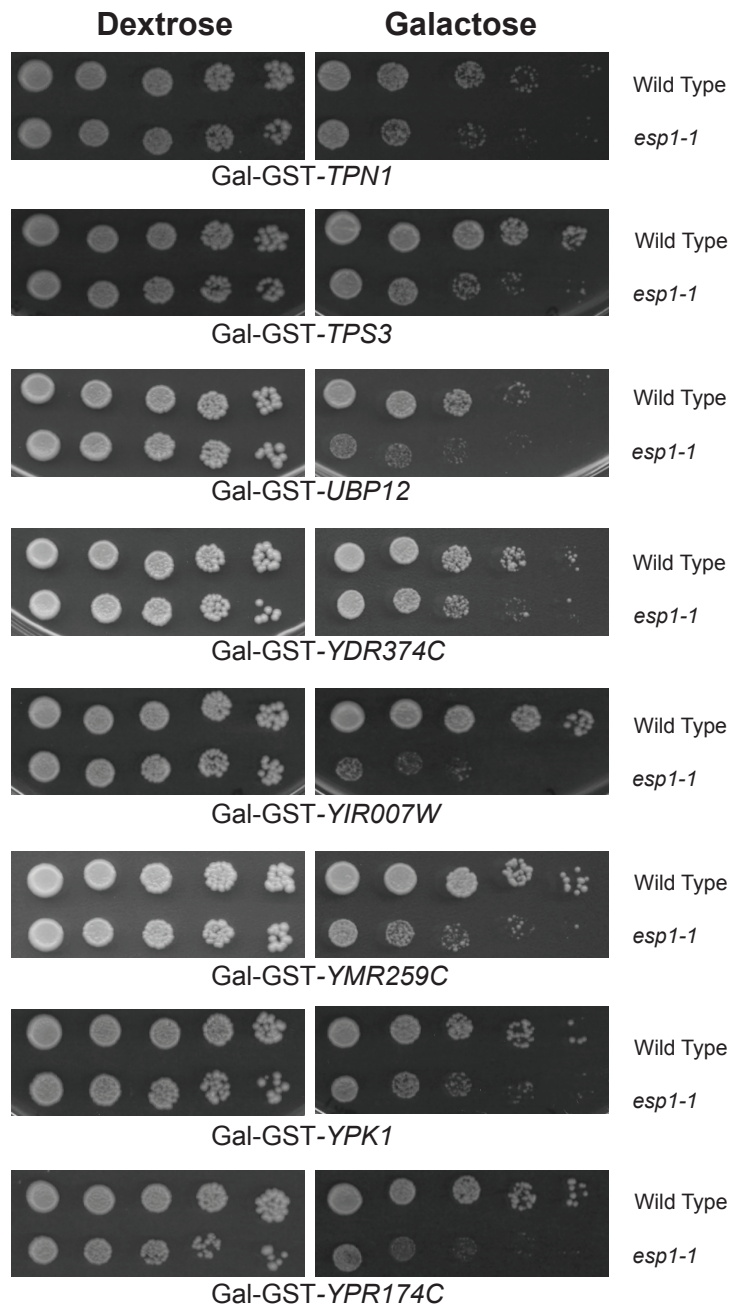

Supplement: S1 Fig — Plasmids of interest were transformed into both WT and esp1-1 strains. Logarithmically growing cells were serially diluted and plated onto dextrose (control) or galactose containing media to confirm growth defect when overexpressed in esp1-1. (PDF) [file pgen.1005109.s001.pdf]

Figure S2

A

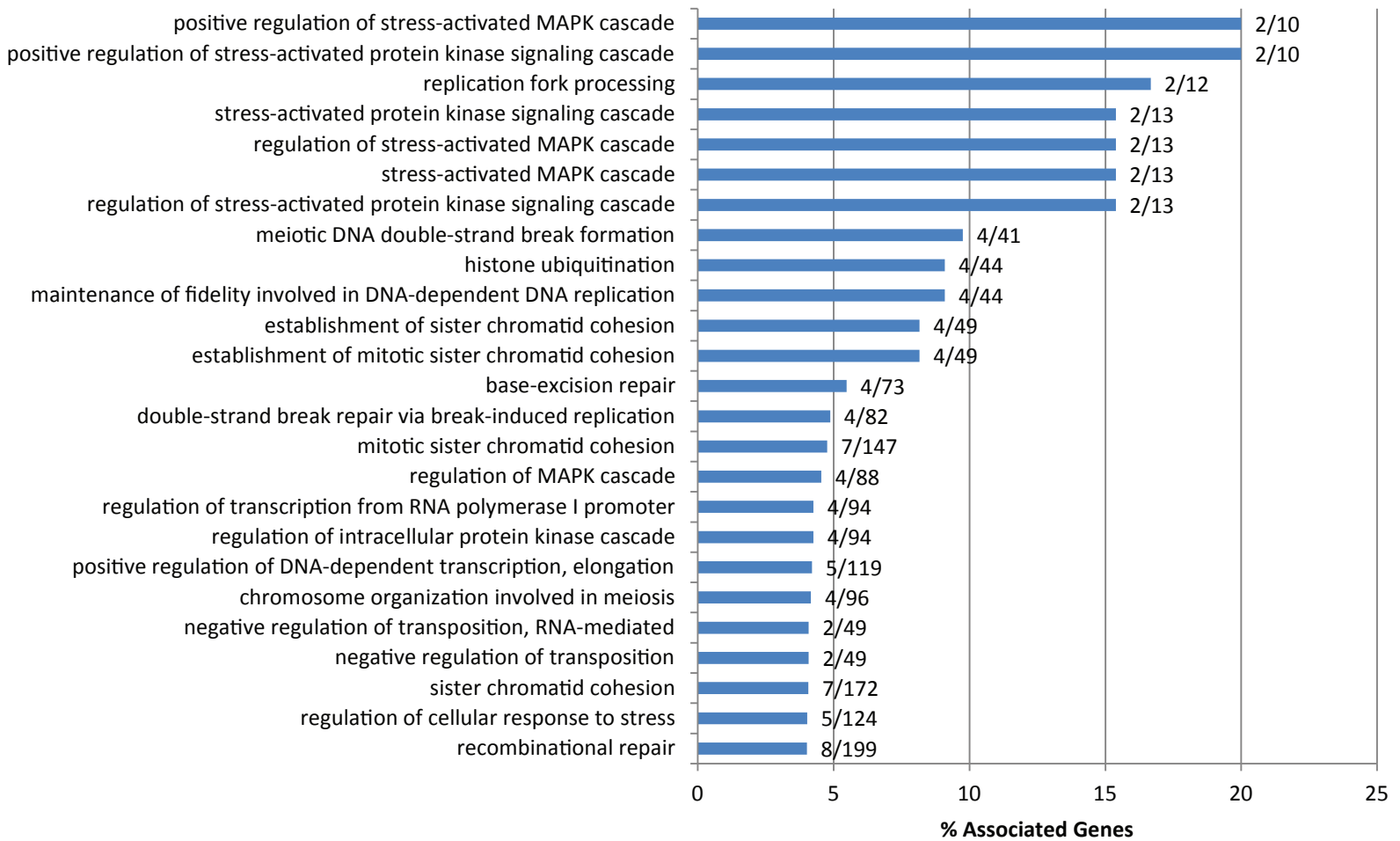

B

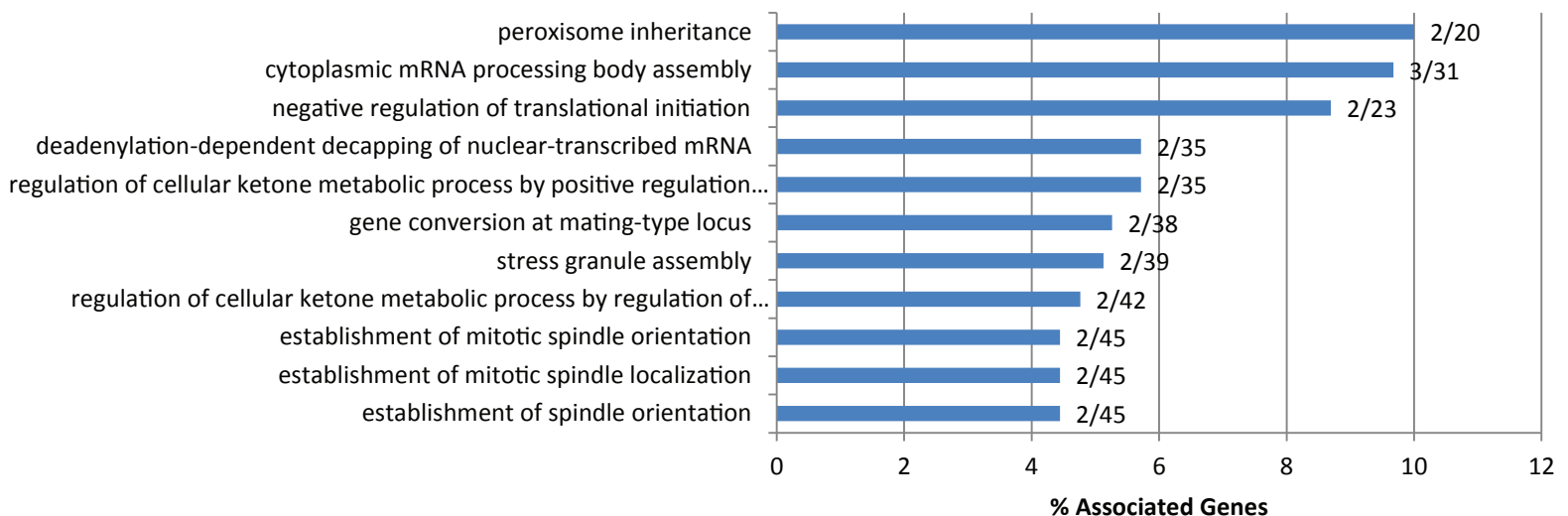

Supplement: S2 Fig — GO terms were clustered using the ClueGO [36] plugin for Cytoscape [35] using a Kappa value of 0.3. For each term, the number of genes found in the corresponding screen are shown relative to the total number of genes assigned to that term. (A) SL screen interactions, (B) SDL screen interactions. (PDF) [file pgen.1005109.s002.pdf]
