# Supplementary material for: A Role for the Budding Yeast Separase, Esp1, in Ty1 Element Retrotransposition
Source: PLoS Genet. 2015 Mar 30;11(3):e1005109. doi: 10.1371/journal.pgen.1005109 (PMC4378997; doi:10.1371/journal.pgen.1005109)
Supplement: S1 Table — (DOCX) [file pgen.1005109.s003.docx]

Table S1. Genes identified in *esp1-1* SL screen

| **ORF** | **Gene Name** | **Ratio (∆∆/∆)** |
| --- | --- | --- |
| *YNL147W* | *LSM7* | 0.047 |
| *YAR002W* | *NUP60* | 0.052 |
| *YGL071W* | *AFT1* | 0.056 |
| *YAL024C* | *LTE1* | 0.062 |
| *YMR186W* | *HSC82* | 0.067 |
| *YMR198W* | *CIK1* | 0.069 |
| *YJL124C* | *LSM1* | 0.072 |
| *YPR141C* | *KAR3* | 0.075 |
| *YHR021C* | *RPS27B* | 0.083 |
| *YLR345W* | *YLR345W* | 0.085 |
| *YML112W* | *CTK3* | 0.096 |
| *YNL064C* | *YDJ1* | 0.103 |
| *YAL021C* | *CCR4* | 0.111 |
| *YPR119W* | *CLB2* | 0.114 |
| *YJL006C* | *CTK2* | 0.126 |
| *YGR020C* | *VMA7* | 0.149 |
| *YOR297C* | *TIM18* | 0.151 |
| *YNL250W* | *RAD50* | 0.155 |
| *YHR010W* | *RPL27A* | 0.156 |
| *YOR123C* | *LEO1* | 0.158 |
| *YDR200C* | *VPS64* | 0.16 |
| *YOR097C* | *YOR097C* | 0.168 |
| *YLR320W* | *MMS22* | 0.178 |
| *YBR122C* | *MRPL36* | 0.225 |
| *YEL027W* | *VMA3* | 0.225 |
| *YEL051W* | *VMA8* | 0.228 |
| *YLR193C* | *UPS1* | 0.236 |
| *YGL240W* | *DOC1* | 0.264 |
| *YLR261C* | *VPS63* | 0.274 |
| *YEL065W* | *SIT1* | 0.277 |
| *YGL088W* | *YGL088W* | 0.299 |
| *YIL040W* | *APQ12* | 0.304 |
| *YKL101W* | *HSL1* | 0.304 |
| *YGR219W* | *YGR219W* | 0.313 |
| *YGR171C* | *MSM1* | 0.315 |
| *YPL055C* | *LGE1* | 0.331 |
| *YPL069C* | *BTS1* | 0.345 |
| *YJR118C* | *ILM1* | 0.353 |
| *YER167W* | *BCK2* | 0.355 |
| *YNL277W* | *MET2* | 0.356 |
| *YOR331C* | *YOR331C* | 0.357 |
| *YLR396C* | *VPS33* | 0.361 |
| *YDR369C* | *XRS2* | 0.367 |
| *YDL074C* | *BRE1* | 0.368 |
| *YPL253C* | *VIK1* | 0.369 |
| *YCL007C* | *YCL007C* | 0.373 |
| *YLR312W-A* | *MRPL15* | 0.387 |
| *YDR450W* | *RPS18A* | 0.414 |
| *YLL002W* | *RTT109* | 0.415 |
| *YDR290W* | *YDR290W* | 0.422 |
| *YKR074W* | *AIM29* | 0.425 |
| *YGL244W* | *RTF1* | 0.428 |
| *YPR132W* | *RPS23B* | 0.438 |
| *YOR202W* | *HIS3* | 0.443 |
| *YGL007W* | *BRP1* | 0.448 |
| *YNL296W* | *YNL296W* | 0.448 |
| *YML024W* | *RPS17A* | 0.452 |
| *YDL115C* | *IWR1* | 0.453 |
| *YKL155C* | *RSM22* | 0.453 |
| *YER040W* | *GLN3* | 0.454 |
| *YKR095W* | *MLP1* | 0.463 |
| *YBR181C* | *RPS6B* | 0.474 |
| *YGL217C* | *YGL217C* | 0.479 |
| *YCR076C* | *FUB1* | 0.497 |
| *YLR262C* | *YPT6* | 0.506 |
| *YOR147W* | *MDM32* | 0.515 |
| *YPL239W* | *YAR1* | 0.529 |
| *YBL096C* | *YBL096C* | 0.531 |
| *YKR094C* | *RPL40B* | 0.532 |
| *YNR051C* | *BRE5* | 0.537 |
| *YHR203C* | *RPS4B* | 0.539 |
| *YFL001W* | *DEG1* | 0.547 |
| *YKL191W* | *DPH2* | 0.559 |
| *YNL140C* | *YNL140C* | 0.56 |
| *YCR033W* | *SNT1* | 0.562 |
| *YGL194C* | *HOS2* | 0.565 |
| *YAL026C* | *DRS2* | 0.579 |
| *YPR133W-A* | *TOM5* | 0.584 |
| *YMR098C* | *ATP25* | 0.585 |
| *YDR136C* | *VPS61* | 0.585 |
| *YFL006W* | *YFL006W* | 0.585 |
| *YGL163C* | *RAD54* | 0.587 |
| *YPL157W* | *TGS1* | 0.588 |
| *YGR229C* | *SMI1* | 0.589 |
| *YLR372W* | *SUR4* | 0.596 |
| *YHR060W* | *VMA22* | 0.598 |
| *YCR024C* | *SLM5* | 0.613 |
| *YLR204W* | *QRI5* | 0.614 |
| *YLR386W* | *VAC14* | 0.616 |
| *YML017W* | *PSP2* | 0.617 |
| *YNL281W* | *HCH1* | 0.618 |
| *YGL168W* | *HUR1* | 0.619 |
| *YPR164W* | *MMS1* | 0.619 |
| *YDR296W* | *MHR1* | 0.621 |
| *YNL242W* | *ATG2* | 0.625 |
| *YFL007W* | *BLM10* | 0.627 |
| *YGL014W* | *PUF4* | 0.635 |
| *YER111C* | *SWI4* | 0.636 |
| *YPL018W* | *CTF19* | 0.644 |
| *YMR224C* | *MRE11* | 0.648 |
| *YOL033W* | *MSE1* | 0.65 |
| *YJR043C* | *POL32* | 0.652 |
| *YDR156W* | *RPA14* | 0.653 |
| *YDL101C* | *DUN1* | 0.655 |
| *YGL029W* | *CGR1* | 0.656 |
| *YPR057W* | *BRR1* | 0.658 |
| *YBR078W* | *ECM33* | 0.658 |
| *YOL150C* | *YOL150C* | 0.66 |
| *YDR318W* | *MCM21* | 0.662 |
| *YBR134W* | *YBR134W* | 0.67 |
| *YNL299W* | *TRF5* | 0.681 |
| *YKL027W* | *YKL027W* | 0.681 |
| *YGL094C* | *PAN2* | 0.683 |
| *YKL185W* | *ASH1* | 0.685 |
| *YNL241C* | *ZWF1* | 0.686 |
| *YOL032W* | *OPI10* | 0.688 |
| *YBR150C* | *TBS1* | 0.688 |
| *YPL184C* | *MRN1* | 0.69 |
| *YPR069C* | *SPE3* | 0.69 |
| *YKL184W* | *SPE1* | 0.691 |
| *YIL158W* | *AIM20* | 0.693 |
| *YCL016C* | *DCC1* | 0.694 |
| *YAR018C* | *KIN3* | 0.694 |
| *YDR181C* | *SAS4* | 0.695 |
| *YBR269C* | *FMP21* | 0.696 |
| *YNL053W* | *MSG5* | 0.696 |
| *YER089C* | *PTC2* | 0.696 |
| *YFL036W* | *RPO41* | 0.696 |
| *YGR052W* | *FMP48* | 0.697 |
| *YGR049W* | *SCM4* | 0.698 |
| *YKL117W* | *SBA1* | 0.699 |
| *YIL136W* | *OM45* | 0.702 |
| *YOR235W* | *IRC13* | 0.703 |
| *YLR110C* | *CCW12* | 0.708 |
| *YPL017C* | *IRC15* | 0.709 |
| *YML129C* | *COX14* | 0.716 |
| *YDL121C* | *YDL121C* | 0.716 |
| *YMR286W* | *MRPL33* | 0.719 |
| *YLR335W* | *NUP2* | 0.72 |
| *YHL023C* | *NPR3* | 0.721 |
| *YNL069C* | *RPL16B* | 0.721 |
| *YPL183W-A* | *RTC6* | 0.721 |
| *YBR035C* | *PDX3* | 0.726 |
| *YGR051C* | *YGR051C* | 0.733 |
| *YDR254W* | *CHL4* | 0.734 |
| *YMR238W* | *DFG5* | 0.745 |
| *YKL156W* | *RPS27A* | 0.758 |
| *YOR314W* | *YOR314W* | 0.758 |
| *YKR059W* | *TIF1* | 0.76 |
| *YDR285W* | *ZIP1* | 0.761 |
| *YBR229C* | *ROT2* | 0.763 |
| *YPL125W* | *KAP120* | 0.769 |
| *YGR012W* | *YGR012W* | 0.769 |
| *YJR056C* | *YJR056C* | 0.77 |
| *YBR297W* | *MAL33* | 0.771 |
| *YBR068C* | *BAP2* | 0.774 |
| *YDR281C* | *PHM6* | 0.775 |
| *YGR183C* | *QCR9* | 0.775 |
| *YGL221C* | *NIF3* | 0.778 |
| *YJR037W* | *YJR037W* | 0.783 |
| *YKL183W* | *LOT5* | 0.794 |
