# Supplementary material for: A Role for the Budding Yeast Separase, Esp1, in Ty1 Element Retrotransposition
Source: PLoS Genet. 2015 Mar 30;11(3):e1005109. doi: 10.1371/journal.pgen.1005109 (PMC4378997; doi:10.1371/journal.pgen.1005109)
Supplement: S2 Table — (DOCX) [file pgen.1005109.s004.docx]

Table S2. Genes identified in *esp1-1* SDL screen

| **ORF** | **Gene name** | **SDL/SDS** | | |
| --- | --- | --- | --- | --- |
| *YLR304C* | *ACO1* | SDL | | |
| *YGR061C* | *ADE6* | SDL | | |
| *YJL194W* | *CDC6* | SDS | | |
| *YMR198W* | *CIK1* | SDL | | |
| *YDL145C* | *COP1* | SDS | | |
| *YDR430C* | *CYM1* | SDS | | |
| *YDL160C* | *DHH1* | SDL | | |
| *YDR440W* | *DOT1* | SDS | | |
| *YBR180W* | *DTR1* | SDS | | |
| *YDR206W* | *EBS1* | SDL | | |
| *YEL030W* | *ECM10* | SDL | | |
| *YDR385W* | *EFT2* | SDL | | |
| *YHR089C* | *GAR1* | SDL | | |
| *YOR185C* | *GSP2* | SDL | | |
| *YJL110C* | *GZF3* | SDS | | |
| *YOL123W* | *HRP1* | SDL | | |
| *YKL201C* | *MNN4* | SDL | | |
| *YOR326W* | *MYO2* | SDS | | |
| *YGL094C* | *PAN2* | SDL | | |
| *YCR077C* | *PAT1* | SDL | | |
| *YGL013C* | *PDR1* | SDS | | |
| *YDL065C* | *PEX19* | SDS | | |
| *YGR077C* | *PEX8* | SDS | | |
| *YCL057W* | *PRD1* | SDL | | |
| *YKL113C* | *RAD27* | SDS | | |
| *YPL153C* | *RAD53* | SDL | | |
| *YDR028C* | *REG1* | SDS | | |
| *YHR056C* | *RSC30* | SDL | | |
| *YPR129W* | *SCD6* | SDL | | |
| *YMR190C* | *SGS1* | SDS | | |
| *YJL089W* | *SIP4* | SDL | | |
| *YOR195W* | *SLK19* | SDL | | |
| *YJL127C* | *SPT10* | SDS | | |
| *YKL020C* | *SPT23* | SDS | | |
| *YOR212W* | *STE4* | SDS | | |
| *YKR010C* | *TOF2* | SDL | | |
| *YGL186C* | *TPN1* | SDS | | |
| *YMR261C* | *TPS3* | SDS | | |
| *YJL197W* | *UBP12* | SDL | | |
| *YDR374C* | YDR374C | SDS | | |
| *YIR007W* | YIR007W | | SDL |  |
| *YMR259C* | YMR259C | | SDS |  |
| *YPK1* | YKL126W | | SDS |  |
| *YPR174C* | YPR174C | | SDL |  |
