# Supplementary material for: A Role for the Budding Yeast Separase, Esp1, in Ty1 Element Retrotransposition
Source: PLoS Genet. 2015 Mar 30;11(3):e1005109. doi: 10.1371/journal.pgen.1005109 (PMC4378997; doi:10.1371/journal.pgen.1005109)
Supplement: S3 Table — (DOCX) [file pgen.1005109.s005.docx]

**Table S3.** Affinity mass spectrometry peptides identified for Esp1 purification

| **ORF** | **Gene Name** | **Protein Score** | **Peptides Identified** | **Sequence Coverage (%)** | **Peptides Identified in mock** |
| --- | --- | --- | --- | --- | --- |
| *YNR016C* | *ACC1* | 1020 | 46 | 17 | 10 |
| *YGL195W* | *GCN1* | 799 | 35 | 10.7 | 0 |
| *YMR229C* | *RRP5* | 392 | 32 | 13.9 | 6 |
| ***YGR098C*** | ***ESP1*** | **546** | **29** | **14.3** | **0** |
| *YPL160W* | *CDC60* | 738 | 29 | 23.5 | 6 |
| *YJL034W* | *KAR2* | 608 | 29 | 32.4 | 7 |
| *YBR079C* | *RPG1* | 451 | 22 | 21.6 | 5 |
| *YOR335C* | *ALA1* | 522 | 21 | 20.9 | 2 |
| *YBL076C* | *ILS1* | 571 | 21 | 17.1 | 5 |
| *YLR153C* | *ACS2* | 318 | 20 | 16 | 5 |
| *YDR127W* | *ARO1* | 313 | 19 | 11.4 | 4 |
| *YER036C* | *ARB1* | 773 | 18 | 22 | 3 |
| *YGL206C* | *CHC1* | 296 | 18 | 8.7 | 4 |
| *YPL226W* | *NEW1* | 316 | 17 | 15.7 | 0 |
| *YDR037W* | *KRS1* | 314 | 17 | 18.8 | 3 |
| *YHR020W* | *YHR020W* | 349 | 16 | 28.6 | 0 |
| *YMR012W* | *CLU1* | 370 | 16 | 13 | 0 |
| ***YDR113C*** | ***PDS1*** | **359** | **16** | **32.4** | **0** |
| *YOR204W* | *DED1* | 319 | 16 | 21.9 | 2 |
| *YIL018W* | *RPL2B* | 381 | 16 | 35.8 | 3 |
| *YGL173C* | *KEM1* | 272 | 15 | 8.3 | 0 |
| *YDL171C* | *GLT1* | 308 | 15 | 5.8 | 3 |
| *YGR264C* | *MES1* | 261 | 15 | 14.6 | 3 |
| *YKL210W* | *UBA1* | 208 | 14 | 12 | 3 |
| *YOR153W* | *PDR5* | 277 | 13 | 8.5 | 0 |
| *YLR342W* | *FKS1* | 354 | 12 | 8.1 | 0 |
| *YER091C* | *MET6* | 175 | 12 | 16.7 | 0 |
| *YGL137W* | *SEC27* | 253 | 12 | 10.7 | 2 |
| *YPL131W* | *RPL5* | 194 | 12 | 28.3 | 2 |
| *YGR285C* | *ZUO1* | 231 | 12 | 17.1 | 2 |
| *YOR341W* | *RPA190* | 189 | 11 | 5.9 | 0 |
| *YDL145C* | *COP1* | 111 | 11 | 10 | 0 |
| *YOR168W* | *GLN4* | 163 | 11 | 13.3 | 0 |
| *YDR238C* | *SEC26* | 215 | 11 | 11.6 | 0 |
| *YOR361C* | *PRT1* | 151 | 10 | 13 | 0 |
| *YHR027C* | *RPN1* | 160 | 10 | 9.9 | 2 |
| *YGL207W* | *SPT16* | 200 | 10 | 11 | 2 |
| *YIL041W* | *GVP36* | 208 | 10 | 30.1 | 2 |
| *YMR108W* | *ILV2* | 164 | 10 | 13.5 | 2 |
| *YHR047C* | *AAP1* | 166 | 9 | 8.9 | 0 |
| *YGR061C* | *ADE6* | 150 | 9 | 8 | 0 |
| *YGL234W* | *ADE5,7* | 176 | 9 | 11.3 | 2 |
| *YEL046C* | *GLY1* | 185 | 9 | 22.5 | 2 |
| *YBR196C* | *PGI1* | 125 | 8 | 18.6 | 0 |
| *YDR234W* | *LYS4* | 271 | 8 | 16.7 | 0 |
| *YDR341C* | *YDR341C* | 81 | 8 | 11.9 | 0 |
| *YKR001C* | *VPS1* | 117 | 8 | 13.1 | 0 |
| *YNL104C* | *LEU4* | 74 | 8 | 11 | 0 |
| *YNL287W* | *SEC21* | 215 | 8 | 9.4 | 0 |
| *YHR183W* | *GND1* | 137 | 8 | 16.4 | 2 |
| *YGR162W* | *TIF4631* | 233 | 7 | 10.2 | 0 |
| *YIL075C* | *RPN2* | 75 | 7 | 5.6 | 0 |
| *YBR084W* | *MIS1* | 157 | 7 | 8.3 | 0 |
| *YDL084W* | *SUB2* | 135 | 7 | 14.3 | 0 |
| *YER086W* | *ILV1* | 88 | 7 | 15.6 | 0 |
| *YFR009W* | *GCN20* | 104 | 7 | 9.8 | 0 |
| *YEL031W* | *SPF1* | 135 | 6 | 3.7 | 0 |
| *YLR106C* | *MDN1* | 178 | 6 | 1.5 | 0 |
| *YDL143W* | *CCT4* | 174 | 6 | 12.5 | 0 |
| *YER025W* | *GCD11* | 135 | 6 | 13.3 | 0 |
| *YGL150C* | *INO80* | 51 | 6 | 1.1 | 0 |
| *YGL210W* | *YPT32* | 62 | 6 | 33.8 | 0 |
| *YGR185C* | *TYS1* | 76 | 6 | 12.7 | 0 |
| *YMR080C* | *NAM7* | 41 | 6 | 3.1 | 0 |
| *YOR046C* | *DBP5* | 133 | 6 | 9.5 | 0 |
| *YGL120C* | *PRP43* | 160 | 6 | 9 | 0 |
| *YGR175C* | *ERG1* | 136 | 6 | 9.7 | 0 |
| *YLR100W* | *ERG27* | 31 | 6 | 12.4 | 0 |
| *YPR181C* | *SEC23* | 168 | 6 | 5.6 | 0 |
| *YGR218W* | *CRM1* | 80 | 5 | 4.4 | 0 |
| *YKL145W* | *RPT1* | 151 | 5 | 9.2 | 0 |
| *YNL132W* | *KRE33* | 114 | 5 | 4 | 0 |
| *YBR143C* | *SUP45* | 150 | 5 | 8.7 | 0 |
| *YCL018W* | *LEU2* | 31 | 5 | 15.1 | 0 |
| *YCL040W* | *GLK1* | 62 | 5 | 6.6 | 0 |
| *YCR009C* | *RVS161* | 55 | 5 | 18.1 | 0 |
| *YDL140C* | *RPO21* | 58 | 5 | 2.3 | 0 |
| *YER031C* | *YPT31* | 51 | 5 | 24.7 | 0 |
| *YER062C* | *HOR2* | 122 | 5 | 10 | 0 |
| *YFR010W* | *UBP6* | 62 | 5 | 13.4 | 0 |
| *YGR116W* | *SPT6* | 68 | 5 | 2.4 | 0 |
| *YKR095W* | *MLP1* | 84 | 5 | 2.3 | 0 |
| *YLR058C* | *SHM2* | 100 | 5 | 10.4 | 0 |
| *YLR447C* | *VMA6* | 95 | 5 | 11 | 0 |
| *YBR080C* | *SEC18* | 59 | 4 | 4.6 | 0 |
| *YDR382W* | *RPP2B* | 195 | 4 | 60.9 | 0 |
| *YMR241W* | *YHM2* | 245 | 4 | 7 | 0 |
| *YFR002W* | *NIC96* | 51 | 4 | 4.3 | 0 |
| *YBL022C* | *PIM1* | 122 | 4 | 3.8 | 0 |
| *YBR145W* | *ADH5* | 75 | 4 | 2.3 | 0 |
| *YDL147W* | *RPN5* | 70 | 4 | 8.5 | 0 |
| *YDR101C* | *ARX1* | 55 | 4 | 5.1 | 0 |
| *YDR170C* | *SEC7* | 68 | 4 | 1.8 | 0 |
| *YER070W* | *RNR1* | 109 | 4 | 5.2 | 0 |
| *YER125W* | *RSP5* | 51 | 4 | 6.8 | 0 |
| *YFL004W* | *VTC2* | 61 | 4 | 5.8 | 0 |
| *YHR137W* | *ARO9* | 53 | 4 | 6.2 | 0 |
| *YHR179W* | *OYE2* | 75 | 4 | 10.5 | 0 |
| *YIR006C* | *PAN1* | 57 | 4 | 2.8 | 0 |
| *YJL050W* | *MTR4* | 93 | 4 | 3.9 | 0 |
| *YJR064W* | *CCT5* | 92 | 4 | 6.9 | 0 |
| *YKL009W* | *MRT4* | 82 | 4 | 20.3 | 0 |
| *YKL104C* | *GFA1* | 97 | 4 | 7.3 | 0 |
| *YLR303W* | *MET17* | 116 | 4 | 9.9 | 0 |
| *YLR384C* | *IKI3* | 48 | 4 | 3 | 0 |
| *YMR038C* | *CCS1* | 59 | 4 | 9.2 | 0 |
| *YMR125W* | *STO1* | 78 | 4 | 4.9 | 0 |
| *YOR108W* | *LEU9* | 74 | 4 | 6 | 0 |
| *YBL039C* | *URA7* | 159 | 4 | 6.6 | 0 |
| *YBR039W* | *ATP3* | 113 | 4 | 15.4 | 0 |
| *YBR221C* | *PDB1* | 58 | 4 | 13.4 | 0 |
| *YDR211W* | *GCD6* | 82 | 4 | 4.5 | 0 |
| *YDR406W* | *PDR15* | 81 | 4 | 1.9 | 0 |
| *YER178W* | *PDA1* | 44 | 4 | 7.4 | 0 |
| *YPR041W* | *TIF5* | 68 | 4 | 9.1 | 0 |
| *YDR381W* | *YRA1* | 99 | 3 | 14.2 | 0 |
| *YKL029C* | *MAE1* | 140 | 3 | 7 | 0 |
| *YKL120W* | *OAC1* | 74 | 3 | 10.8 | 0 |
| *YML123C* | *PHO84* | 31 | 3 | 6.6 | 0 |
| *YPR019W* | *MCM4* | 25 | 3 | 1.7 | 0 |
| *YCR057C* | *PWP2* | 34 | 3 | 2.7 | 0 |
| *YCR084C* | *TUP1* | 109 | 3 | 4.6 | 0 |
| *YDL100C* | *GET3* | 36 | 3 | 7.9 | 0 |
| *YDR129C* | *SAC6* | 187 | 3 | 5.8 | 0 |
| *YDR158W* | *HOM2* | 63 | 3 | 5.8 | 0 |
| *YDR353W* | *TRR1* | 144 | 3 | 15.7 | 0 |
| *YER003C* | *PMI40* | 27 | 3 | 9.1 | 0 |
| *YER052C* | *HOM3* | 41 | 3 | 6.6 | 0 |
| *YGL026C* | *TRP5* | 98 | 3 | 5.9 | 0 |
| *YGR229C* | *SMI1* | 69 | 3 | 5.9 | 0 |
| *YGR256W* | *GND2* | 79 | 3 | 6.7 | 0 |
| *YHR098C* | *SFB3* | 105 | 3 | 3.9 | 0 |
| *YIL109C* | *SEC24* | 57 | 3 | 3.8 | 0 |
| *YJL033W* | *HCA4* | 27 | 3 | 3.4 | 0 |
| *YKL126W* | *YPK1* | 90 | 3 | 5.7 | 0 |
| *YKL216W* | *URA1* | 33 | 3 | 4.8 | 0 |
| *YLL018C* | *DPS1* | 53 | 3 | 6.5 | 0 |
| *YLR196W* | *PWP1* | 73 | 3 | 4.5 | 0 |
| *YNL163C* | *RIA1* | 31 | 3 | 2.4 | 0 |
| *YNL220W* | *ADE12* | 66 | 3 | 5.3 | 0 |
| *YNL231C* | *PDR16* | 26 | 3 | 9.4 | 0 |
| *YNL241C* | *ZWF1* | 100 | 3 | 7.5 | 0 |
| *YNL307C* | *MCK1* | 48 | 3 | 5.9 | 0 |
| *YOR151C* | *RPB2* | 53 | 3 | 2.9 | 0 |
| *YOR165W* | *SEY1* | 51 | 3 | 3.5 | 0 |
| *YPL043W* | *NOP4* | 58 | 3 | 5 | 0 |
| *YPL058C* | *PDR12* | 66 | 3 | 1.5 | 0 |
| *YPL190C* | *NAB3* | 28 | 3 | 3.5 | 0 |
| *YPL242C* | *IQG1* | 19 | 3 | 0.7 | 0 |
| *YDL058W* | *USO1* | 53 | 3 | 1.5 | 0 |
| *YIL043C* | *CBR1* | 34 | 3 | 9.9 | 0 |
| *YPL119C* | *DBP1* | 0 | 3 | 2.9 | 0 |
