# Supplementary material for: A Role for the Budding Yeast Separase, Esp1, in Ty1 Element Retrotransposition
Source: PLoS Genet. 2015 Mar 30;11(3):e1005109. doi: 10.1371/journal.pgen.1005109 (PMC4378997; doi:10.1371/journal.pgen.1005109)
Supplement: S4 Table — (DOCX) [file pgen.1005109.s006.docx]

**Table S4. Yeast strains used in this study**

| **Strain** | **Genotype** | **Source** |
| --- | --- | --- |
| Y7092 | Mat α *can1∆::STE2pr-Sp_his5 lyp1∆ his3∆1 leu2∆0 ura3∆0 met15∆0* | [1] |
| BY4741 | MAT a *his3∆1 leu2∆0 ura3∆0 met15∆0* | [2] |
| YKH64 | Mat α *can1∆::STE2pr-Sp_his5 lyp1∆ his3∆1 leu2∆0 ura3∆0 met15∆0 esp1-1::natR* | This study |
| YKH186 | Mat a *ura3-52 lys2-801_amber ade2-101 trp1-*Δ*63 his3-*Δ*200 leu2-*Δ*1* Esp1-13myc::KanMX6 | This study |
| YKH558 | *MAT*a *his3*Δ*1 leu2*Δ*0 lys2*Δ*0 met15*Δ *ura3*Δ*0 pds1-128::KanMX6* | [3] |
| YKH559 | *MAT*a *his3*Δ*1 leu2*Δ*0 lys2*Δ*0 met15*Δ *ura3*Δ*0 scc2-4::KanMX6* | [3] |
| YKH560 | *MAT*a *his3*Δ*1 leu2*Δ*0 lys2*Δ*0 met15*Δ *ura3*Δ*0 scc1-73::KanMX6* | [3] |
| YKH580 | *MAT*a *his3*Δ*1 leu2*Δ*0 lys2*Δ*0 met15*Δ *ura3*Δ*0 scc3-1::KanMX6* | [3] |
| YKH582 | *MAT*a *his3*Δ*1 leu2*Δ*0 lys2*Δ*0 met15*Δ *ura3*Δ*0 smc1-2::KanMX6* | [3] |
| YKH583 | *MAT*a *his3*Δ*1 leu2*Δ*0 lys2*Δ*0 met15*Δ *ura3*Δ*0 smc1-259::KanMX6* | [3] |
| YKH584 | *MAT*a *his3*Δ*1 leu2*Δ*0 lys2*Δ*0 met15*Δ *ura3*Δ*0 smc3-1::KanMX6* | [3] |
| YKH585 | *MAT*a *his3*Δ*1 leu2*Δ*0 lys2*Δ*0 met15*Δ *ura3*Δ*0 smc3-42::KanMX6* | [3] |
| YKH613 | Mat α *can1∆::STE2pr-Sp_his5 lyp1∆ his3∆1 leu2∆0 ura3∆0 met15∆0 esp1-1::natR scc1-73::KanMX6* | This study |
| YKH638 | *MAT*a *his3*Δ*1 leu2*Δ*0 lys2*Δ*0 met15*Δ *ura3*Δ*0 spt3::KanMX6* | [4] |
| YKH757 | *MAT*a *his3*Δ*1 leu2*Δ*0 lys2*Δ*0 met15*Δ *ura3*Δ*0 SCC1-GFP::HIS3* | [5] |
| YKH761 | *MAT*a *his3*Δ*1 leu2*Δ*0 lys2*Δ*0 met15*Δ *ura3*Δ*0 ESP1-GFP::HIS3* | [5] |
| YLM2071 | *MAT*a *his3*Δ*1 leu2*Δ*0 lys2*Δ*0 met15*Δ *ura3*Δ*0 PDS1-GFP::HIS3* | This study |
| YKH900 | *MAT*a *his3*Δ*1 leu2*Δ*0 lys2*Δ*0 met15*Δ *ura3*Δ*0 NDC80-GFP::HIS3* | [5] |
| YM2407 | *MAT*a *URA::CMV-tTA MATa his3-1 leu2-0met15- Kan*  *-tetO7-PDS1* | [6] |
| YLM2089 | *MAT*a *Kan-TetO_7_-PDS1 ESP1-GFP::HIS3* | This study |
| YM2377 | *MATa/α esp1ΔhphMX4/ESP1* *his3*Δ*1/ his3*Δ*1 leu2*Δ*0/ leu2*Δ*0 lys2*Δ*0/LYS2 met15*Δ*/MET15 ura3*Δ*0/ ura3*Δ*0* | This study |
| YM2390 | Mata esp1ΔhphMX4 *his3*Δ*1 leu2*Δ*0 ura3*Δ*0* *pesp1n122-LEU2-CEN* | This study |
| YM2392 | *Mata esp1ΔhphMX4 his3*Δ*1 leu2*Δ*0 ura3*Δ*0* *pesp1b120-LEU2-CEN* | This study |
| YM2396 | *Mata esp1ΔhphMX4* *his3*Δ*1 leu2*Δ*0 ura3*Δ*0* *pESP1-LEU2-CEN* | This study |
| YM2400 | *Mata esp1ΔhphMX4* *his3*Δ*1 leu2*Δ*0 ura3*Δ*0* *pesp1c113-LEU2-CEN* | This study |
| YM2410 | *MAT*a *lyp1∆ his3∆1 leu2∆0 ura3∆0 met15∆0 esp1-1::natR* | This study |
| YSM3 | *MAT*a *his3*Δ*1 leu2*Δ*0 lys2*Δ*0 met15*Δ *ura3*Δ*0 cdc14-1::KanMX6* | [3] |
| YSM1 | *MAT*a *his3*Δ*1 leu2*Δ*0 lys2*Δ*0 met15*Δ *ura3*Δ*0 cdc5-1::KanMX6* | [3] |
|  | *MAT*a *his3*Δ*1 leu2*Δ*0 lys2*Δ*0 met15*Δ *ura3*Δ*0 cdc15-2:KanMX6* | [3] |
|  | *MAT*a *his3*Δ*1 leu2*Δ*0 lys2*Δ*0 met15*Δ *ura3*Δ*0 dbf2-1::KanMX6* | [3] |
|  | *MAT*a *his3*Δ*1 leu2*Δ*0 lys2*Δ*0 met15*Δ *ura3*Δ*0 spo12ΔKanMX6* | [4] |
|  | *MAT*a *his3*Δ*1 leu2*Δ*0 lys2*Δ*0 met15*Δ *ura3*Δ*0 slk19ΔKanMX6* | [4] |
|  |  |  |

**References**

1. Tong AH, Lesage G, Bader GD, Ding H, Xu H, et al. (2004) Global mapping of the yeast genetic interaction network. Science 303: 808-813.

2. Brachmann CB, Davies A, Cost GJ, Caputo E, Li J, et al. (1998) Designer deletion strains derived from Saccharomyces cerevisiae S288C: a useful set of strains and plasmids for PCR-mediated gene disruption and other applications. Yeast 14: 115-132.

3. Li Z, Vizeacoumar FJ, Bahr S, Li J, Warringer J, et al. (2011) Systematic exploration of essential yeast gene function with temperature-sensitive mutants. Nat Biotechnol 29: 361-367.

4. Giaever G, Chu AM, Ni L, Connelly C, Riles L, et al. (2002) Functional profiling of the Saccharomyces cerevisiae genome. Nature 418: 387-391.

5. Huh WK, Falvo JV, Gerke LC, Carroll AS, Howson RW, et al. (2003) Global analysis of protein localization in budding yeast. Nature 425: 686-691.

6. Mnaimneh S, Davierwala AP, Haynes J, Moffat J, Peng WT, et al. (2004) Exploration of essential gene functions via titratable promoter alleles. Cell 118: 31-44.
